# Root Proteomics Reveals the Effects of Wood Vinegar on Wheat Growth and Subsequent Tolerance to Drought Stress

**DOI:** 10.3390/ijms20040943

**Published:** 2019-02-21

**Authors:** Yuying Wang, Ling Qiu, Qilu Song, Shuping Wang, Yajun Wang, Yihong Ge

**Affiliations:** 1 College of Agronomy, Northwest A&F University, Yangling 712100, China; wyy0624@126.com (Y.W.); songqilu1234@163.com (Q.S.); 2 Northwest Research Center of Rural Renewable Energy, Exploitation and Utilization of Ministry of Agriculture, Northwest A&F University, Yangling 712100, China; wangyajunkaka@outlook.com; 3 College of Mechanical and Electronic Engineering, Northwest A&F University, Yangling 712100, China; 4 Hubei Key Laboratory of Waterlogging Disaster and Agricultural Use of Wetland, College of Agronomy, Yangtze University, Jingzhou 434025, China; wangshuping2003@126.com; 5 Biogas Institute of Ministry of Agriculture and Rural Affairs, Chinese Academy of Agricultural Science, Chengdu 610041, China; geyihong@caas.cn

**Keywords:** wheat, root, wood vinegar, drought stress, ROS, ABA, proteome

## Abstract

Wood vinegar (WV) or pyroligneous acid (PA) is a reddish-brown liquid created during the dry distillation of biomass, a process called pyrolysis. WV contains important biologically active components, which can enhance plant growth and tolerance to drought stress. However, its mechanism of action remains unknown. Our results after presoaking wheat seeds with various concentrations of WV indicate that a 1:900 WV concentration can significantly enhance growth. To investigate the response of wheat roots to drought stress, we compared quantitative proteomic profiles in the roots of wheat plants grown from seeds either presoaked (treatment) or non-presoaked (control) with WV. Our results indicated that the abscisic acid (ABA) content of wheat roots in the WV treatment was significantly increased. Reactive oxygen species (ROS) and malonaldehyde (MDA) levels roots were significantly lower than in the control treatment under drought stress, while the activity of major antioxidant enzymes was significantly increased. Two-dimensional electrophoresis (2D-PAGE) identified 138 differentially accumulated protein (DAP) spots representing 103 unique protein species responding to drought stress in wheat roots of the control and WV-treated groups. These DAPs are mostly involved in the stress response, carbohydrate metabolism, protein metabolism, and secondary metabolism. Proteome profiles showed the DAPs involved in carbohydrate metabolism, stress response, and secondary metabolism had increased accumulation in roots of the WV-treated groups. These findings suggest that the roots from wheat seeds presoaked with WV can initiate an early defense mechanism to mitigate drought stress. These results provide an explanation of how WV enhances the tolerance of wheat plants to drought stress.

## 1. Introduction

Wood vinegar (WV) or pyroligneous acid (PA), a translucent reddish-brown aqueous liquid, is a by-product of the carbonization of tree branches, crop straw, bamboo, wood residue, and other biomaterials [1,2]. WV is a complex mixture, which contains various types of complicated chemical ingredients, namely organic acids, phenolic, alkane, furan derivatives, esters, alcohol, sugar derivatives, and nitrogen compounds [1,3]. The chemical composition of WV mainly depends on the heating rate, temperature, residence time, particle size, and the feedstock [4]. As a natural agricultural material, it contains important biologically active components, such as organic acid and phenolic compounds, and has been widely applied in the areas of medicine, food, and agriculture [5,6]. Most notably in agriculture, WV has been widely utilized as an insect repellent, soil ameliorant, and foliar fertilizer [1,7,8]. Studies indicate that WV improves seed germination rate and accelerates the growth of roots, stems, leaves, flowers, and fruits [2,9]. Further studies have investigated the antioxidant activities of the acids with regard to their radical-scavenging activity and reducing power [10,11]. There has also been an increasing interest in the antioxidant activities of WV and its use in food to replace synthetic antioxidants; however, previous studies based on typical chemical assays have valued the antioxidant for agricultural use only. There are no reports to date that focus on the regulation of the molecular mechanism of WV in plants under stress.

Wheat (*Triticum aestivum* L.) is one of the most important food sources. Demand for wheat is rising continually as a result of population growth and increasing consumption per capita for half a century [12]. However, wheat growth and yield are seriously influenced by drought stress, most notably at the seedling, stem elongation, booting, anthesis, and grain formation stages [13]. Young plants are susceptible to water deficit due to their low biomass, undeveloped protective structure, and water requirements for growth [14]. Roots are the initial receptors that signal a water deficit, followed by a series of responses at the morphological, physiological, and cellular levels. A well-developed root system can assist water uptake during drought conditions [15]. Hence, roots are important for maintaining crop yields, especially when plants are suffering drought stress.

Proteomics has become a powerful tool for creating a proteome profile of plants in response to drought stress [16]. In recent years, several comparative proteomics studies of wheat roots have been undertaken to assess response to drought stress [17,18,19,20]. Studies indicate that proteins related to defense and oxidative stress responses and involved in protein folding, such as heat shock proteins (HSPs), accumulate in greater abundance in wheat, soybean, and rice roots in response to drought stress [18,21,22]. Such increased abundance plays a vital role in scavenging accumulated ROS [23] and preventing aggregation and refolding of non-active proteins. There is evidence that proteins involved in bioenergy metabolism, such as acetyl CoA synthesis or the tricarboxylic acid cycle (TCA), are accumulated in rice roots during drought for increased demand for energy [21]. Meanwhile, proteins involved in cell wall biogenesis, amino acid metabolism, secondary metabolism, and signal transduction show abundant changes in response to drought in plants [18,21,22,24,25]. Exogenous WV pretreatment enhances root growth and tolerance in some plants to subsequent drought stress; however, the mechanisms have remained obscure. In this paper, we investigate the proteome pattern of wheat roots following a WV seed presoaking treatment under drought stress to explore further the molecular mechanisms underlying WV induced drought tolerance.

## 2. Results

### 2.1. Effects of WV Pretreatment on Phenotype and Growth Parameters of Wheat Seedlings

**Figure 1:**
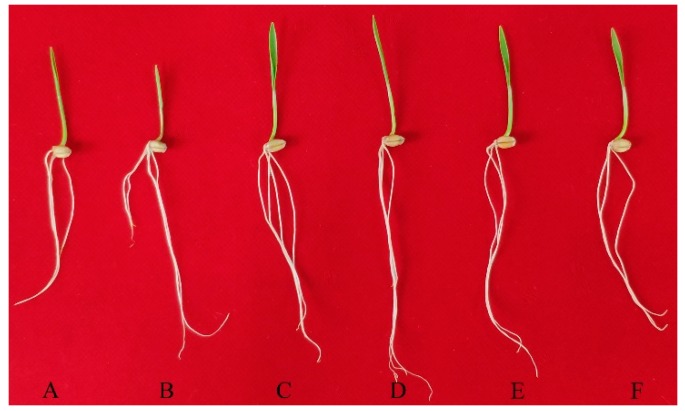
Phenotypic changes in wheat plants following seed soaking treatments with different concentrations of wood vinegar (WV). The various concentrations include 0 (**A**, CK group), 1:300, 1:600, 1:900, 1:1200, 1:1500 (primary WV:ddH_2_O_2_ (*V:V*); **B**–**F**, WV treated groups).

Wheat seeds were soaked in different concentrations of WV (primary WV:ddH_2_O_2_ (*V:V*) = 1:300–1:1500) for 3 days. We found that 1:900 was the optimal ratio for seedling growth and that high concentrations had adverse effects (1:300; Figure 1). Results from fresh weight (FW) and dry weight (DW) of shoots, and roots results were confirmed by quantitative analysis (Table 1, Table 2 and Table S2A). The FW of wheat shoots and roots that had been pretreated with 1:900 WV for 3 days, were significantly higher than those of the control, by 10.0% and 15.7% at 2 days, 15.5% and 38.6% at 3 days, 13.4% and 22.1% at 4 days, 14.9% and 20.8% at 5 days, and 16.5% and 15.9% at 6 days, respectively (Table 1, Table 2, and Table S2A). The DW of wheat shoots and roots that had been pretreated with 1:900 WV for 3 days, were significantly increased by 1.68 and 1.89-fold at 2 days, 1.66 and 2.95-fold at 3 days, 1.34 and 1.92-fold at 4 days, 1.39 and 1.94-fold at 5 days, and 1.47 and 1.94-fold at 6 days, respectively (Table 1, Table 2 and Table S2A). Meanwhile, the total length, surface area, total volume, and mean diameter of the roots from the root system scanning analysis were significantly higher than the control, and there was a maximum promotion at the 1:900 WV concentration (Table S2B, Figure S2C). The height of the shoots pretreated with 1:900 WV for 3 days were significantly greater than those of the control and other WV concentations (Figure 1 and Figure S2B, Table S2C).

### 2.2. Physiological Changes in Wheat Seedlings Under Drought Conditions Following WV Pretreatment

**Figure 2:**
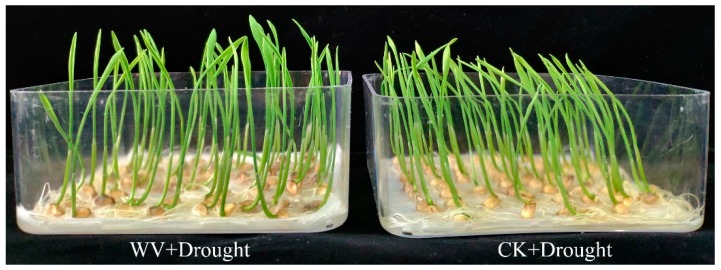
Phenotypic changes of wheat seedlings in both the control and WV treated groups under a drought stress treatment for 2 days. The concentration of WV was 0 and 1:900 in control and WV treated groups, respectively. Drought stress was simulated with PEG 6000(−1 MPa).

To demonstrate the effect of the WV (1:900) seed soaking treatment on drought tolerance, wheat plants of both the control and WV treated groups were exposed to polyethylene glycol (PEG)-induced drought stress (PEG-6000, −1 MPa) for 2 days. Results showed that seedlings in the control group were stunted and wilted; in contrast, WV treated seedlings exhibited less wilting (Figure 2). The FW and DW of shoots were significantly higher than those of the control, by 33.0% and 45.7% at 5 days (the first day after drought stress), and 46.6% and 52.7% at 6 days (the second day after drought stress; Table S2D). The FW and DW of roots were significantly higher than those of the control, by 37.4% and 42.9% at 5 days (the first day after drought stress), and 40.0% and 58.9% at 6 days (the second day after drought stress; Table S2D). The total length, mean diameter, total area, and total volume from root system scanning analysis showed a lower impact of drought in wheat roots of the WV treated groups compared with those of the control (Figure S2D, Table S2E).

**Figure 3:**
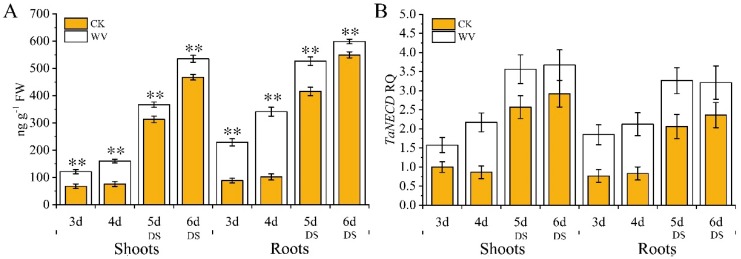
Dynamic changes in abscisic acid (ABA) content and expression pattern analysis of *TaNECD* of wheat shoots and roots in both the control and WV treated groups before and after the drought stress treatments. The concentration of WV was 0 and 1:900 in control and WV treated groups, respectively. The stages of non-drought stress and drought stress were from day 3 to day 4 and from day 5 to day 6, respectively, in the control and WV treated groups. (**A**) Changes of ABA content; (**B**) expression pattern of *TaNECD*. Data are means ± SD of three independent experiments (biological replicates). The significance of differences was assessed by Student’s *t*-test (* *p* < 0.05, ** *p* < 0.01). RQ means relative quantification.

To explore the dynamic changes of the ABA content on drought tolerance, ABA levels in wheat shoots and roots of both the control and the WV treated groups (1:900) were measured before and after drought stress treatments. The ABA content of the roots was higher than in the shoots of both groups (Figure 3A). In the control group, there was no significant change in ABA content in shoots and roots at 3 and 4 days (Figure 3A); however, ABA content was significantly higher in shoots and roots at 5 and 6 days (an increase over day 3 of 4.62 and 6.70-fold in shoots and 4.70 and 5.40-fold in roots, respectively; Figure 3A). Meanwhile, in the WV treated group, ABA content increased from day 3 to day 6 (an increase over day 3 of 1.32, 3.02, and 4.41-fold in shoots, and 1.49, 2.30, and 2.61-fold in roots, respectively; Figure 3A); furthermore, there were significantly higher levels than in the control group (increased by 1.79 and 2.59-fold at 3 days; 2.10, and 3.35-fold at 4 days; 1.17 and 1.26-fold at 5 days; and 1.15 and 1.09-fold at 6 days in shoots and roots, respectively; Figure 3A). Real-time PCR results of 9-cis-epoxycarotenoid dioxygenase gene [*TaNECD*; National Coalition Building Institute (NCBI) accession: KX711890.1], the key gene of ABA biosynthesis, showed the same changing trend as ABA content in both groups (Figure 3B).

**Figure 4:**
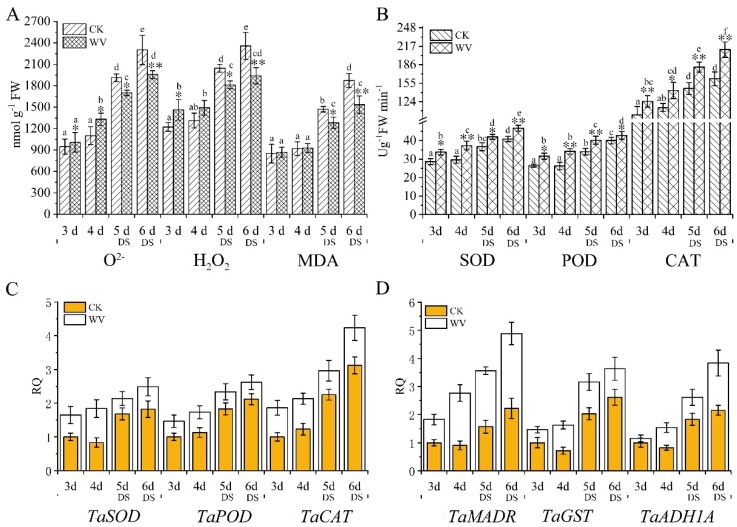
Assessment of ROS content, oxidative stress analysis, and expression pattern analysis of related antioxidative genes in wheat roots in both the control and WV treated groups before and after drought stress. The concentration of WV was 0 and 1:900 in the control and WV treated groups, respectively. The stages of non-drought stress and drought stress were from day 3 to day 4 and from day 5 to day 6, respectively, in both the control and WV treated groups. (**A**) The generation rate of O^2−^ and content of H_2_O_2_ in control wheat roots and WV treated roots; the content of malonaldehyde (MDA) in the control wheat roots and WV treated shoots. (**B**) The activity of superoxide dismutase (SOD), guaiacol peroxidase (POD), and catalase (CAT) in the control wheat roots and WV treated roots. (**C**) Expression pattern analysis of *TaSOD*, *TaPOX1*, and *TaAPX1*. (**D**) Expression pattern analysis of *TaMDAR*, *TaGST*, and *TaADH1A*. Data are means ± SD of three independent experiments (biological replicates). The significance of differences was assessed by Student’s t-test (* *p* < 0.05, ** *p* < 0.01). RQ, relative quantification.

In order to explore whether the generation and accumulation of ROS in both groups of wheat roots before and after drought stress treatment, ROS content and antioxidant enzyme activity were measured. The content of O^2−^ and H_2_O_2_ increased from day 3 and day 6, respectively, in both the control and WV treated groups. The WV roots had higher levels of O^2−^, H_2_O_2_, and MDA than the control group from 3 to 4 days, and content increased by 1.06 and 1.19-fold at 3 days, and 1.21 and 1.14-fold at 4 days, respectively (Figure 4A). Meanwhile, levels of O^2−^ and H_2_O_2_ in the WV treated group decreased by 0.89 and 0.89-fold at 5 days, and 0.85 and 0.82-fold at 6 days, respectively, as compared to the control group (Figure 4A). At the same time, the activities of superoxide dismutase (SOD, EC.1.15.1.1), guaiacol peroxidase (POD, EC1.11.1.7), and catalase (CAT, EC 1.11.1.6) were measured, and the results showed that the WV roots had higher activity of all 3 antioxidant enzymes than did the control group (Figure 4B). These enzymes increased significantly in the WV treated group from day 3 to day 6; there was no significant change in activity of all 3 antioxidant enzymes from day 3 to day 4 in the control group, and their activity increased significantly from day 5 to day 6 under subsequent drought stress (Figure 4B). Quantification of related antioxidant genes performed by real-time PCR, including peroxidase 1 gene (*TaPOX1*, NCBI accession: X85227.1; spot49, EMS54484.1), Cu/Zn superoxide dismutase gene (*TaSOD*, NCBI accession: AK457377), L-ascorbate peroxidase 1 gene (*TaAPX1*, NCBI accession: XM_020316778; spot 108, 110, 111, 112, EMS61931.1), glutathione transferase gene (*TaGST*, NCBI accession: AJ414697; spot 114, CAC94001.1), alcohol dehydrogenase gene (*TaADH1A*, NCBI accession: AK457420 ; spot 56, ABL74258.1), and monodehydroascorbate reductase gene (*TaMDAR*, NCBI accession: KC884831.1; spot 47, EMS50440.1), showed the same results as the antioxidant enzymes (Figure 4C,D). Excessive ROS can oxidize membrane lipids and generate MDA, which can aggravate damage to membrane structure. In order to explore whether the generation and accumulation of MDA caused membrane lipid peroxidation damage, MDA content was measured. No significant difference in MDA concentrations was found between the control and WV treated groups at 3 days and 4 days; however, MDA concentrations were notably higher in the control group than the WV treated group under drought stress (Figure 4A).

### 2.3. Analysis of Differentially Accumulated Protein Spots (DAPs) in Control and WV Pretreated Roots Under Drought Tolerance

To understand the proteome response to short-term drought stress of wheat roots after WV pretreatment, and the changes in proteomes of wheat roots from the control, WV treated groups (1:900; drought stress treatment condition for 2 days) were analyzed by Two-dimensional gel electrophoresis (2-DE). The protein maps produced from three independent biological replicates showed a high reproducibility based on analysis using PDQuest software (Figure S3). PCA analysis indicated the homogeneity of biological replicates and difference of the treatments (Figure S4).

**Figure 5:**
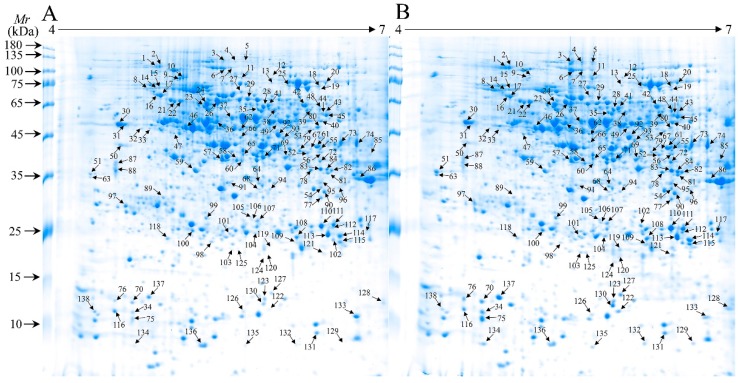
Two-dimensional gel electrophoresis (2-DE) image analysis of proteomes in control and WV treated wheat roots. (**A**) control group; (**B**) WV treated group (1:900).

Figure 5 shows a representative gel image of proteins extracted from the control and WV treated groups. Protein spots [1799 (±97) and 1803 (±26)] were reproducibly detected using PDQuest software from the roots of the control and WV-treated groups, respectively (biological replicates, *n* = 3). From a spot-to-spot comparison and based on statistical analysis, a total of 138 spots (numbered from 1 to 138) exhibited at least a 1.5-fold (Student’s *t*-Test, *p* < 0.05) difference in abundance between the control and WV treated groups (Figure 5, Table S4). In total, 77 spots had a >1.5-fold change in abundance (*p* < 0.05) and 61 spots showed a >2.0-fold change by comparing the two groups; meanwhile, in the roots of the WV treated group, 106 spots exhibited up-regulated expression (53 spots >1.5-fold and 53 spots showed a >2.0-fold change) and 32 spots down-regulated expression (24 spots >1.5-fold and 8 spots showed a >2.0-fold change; Table S4) compared with the control group. In all, 138 differential protein species showed quantitative changes (Figure 5, Table S4). Master gel and several typical examples of DAPs showing different profiles are exhibited in Supplementary Figures S5 and S6.

### 2.4. Identification and Functional Classification of DAPs

A total of 138 DAPs were analyzed by MALDI-TOF/TOF MS and all of them were successfully identified by MS/MS (Table S5). All of the 138 identified protein species were functionally annotated in the current database (Table S5). In summary, 138 identities represented 103 unique protein species.

**Figure 6:**
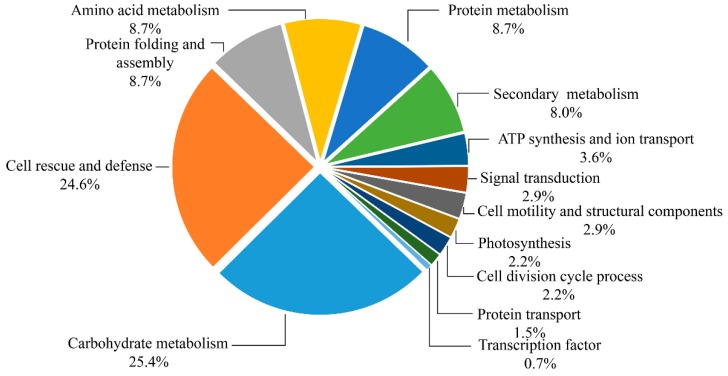
Functional classification of 138 identified proteins. Distribution of proteins according to their biological functions.

Based on the metabolic and functional features of the wheat roots, all of the 138 identified protein species were classified into 13 major categories, including carbohydrate metabolism, cell rescue and defense, protein folding and assembly, amino acid metabolism, protein metabolism, secondary metabolism, ATP synthesis and ion transport, signal transduction, cell motility and structural components, photosynthesis, cell division cycle process, transcription factor, and protein transport (Figure 6). Eighty-four percent of these identified protein species were implicated in the first six functional groups, whereas the largest functional groups that were greatly affected by drought stress were the protein species involved in carbohydrate metabolism and cell rescue and defense (25.4% and 24.6%). Further analysis of the change of abundance in each group revealed that proteins involved in protein folding and assembly (8.7%), amino acid metabolism (8.7%), protein metabolism (8.7%), and secondary metabolism (8.0%) were overrepresented, either in number or in expression level, suggesting that these processes were susceptible to drought stress. In order to visualize the protein expression patterns of all 13 categories, the hierarchical clustering of proteins was analyzed (Figure S7).

In general, the monoisotopic mass (*M*r) calculated by SDS-PAGE with protein standard markers has about a ±10% error compared with the theoretical *M*r value. In our work, 27 identities among all 138 identities had a smaller observed *M*r value than theoretical *M*r value (Table S5). This result indicated these protein species might be partially degraded. Besides, 13 identities among all 138 identities had larger experimental *pI* values than theoretical *pI* values (Table S5), suggesting that these identities may be modified. Some identified protein spots from different positions in the same gel with different observed *M*r and *pI* were found to have the same name and NCBI accession number, whereas these proteins spots should be considered different protein species. For instance, spot 101, 104, and 105 was identified as triosephosphat-isomerase (TPI; CAC14917.1), and spot 110, 111 and 112 were identified as L-ascorbate peroxidase 1 (EMS61931.1). These protein spots were recognized to be different protein species due to gene polymorphisms, alternatively spliced transcripts, proteolytically processed protein species, and PTMs, which might have differential biological function [26].

### 2.5. PPI Analysis of Identified Membrane Proteins

**Figure 7:**
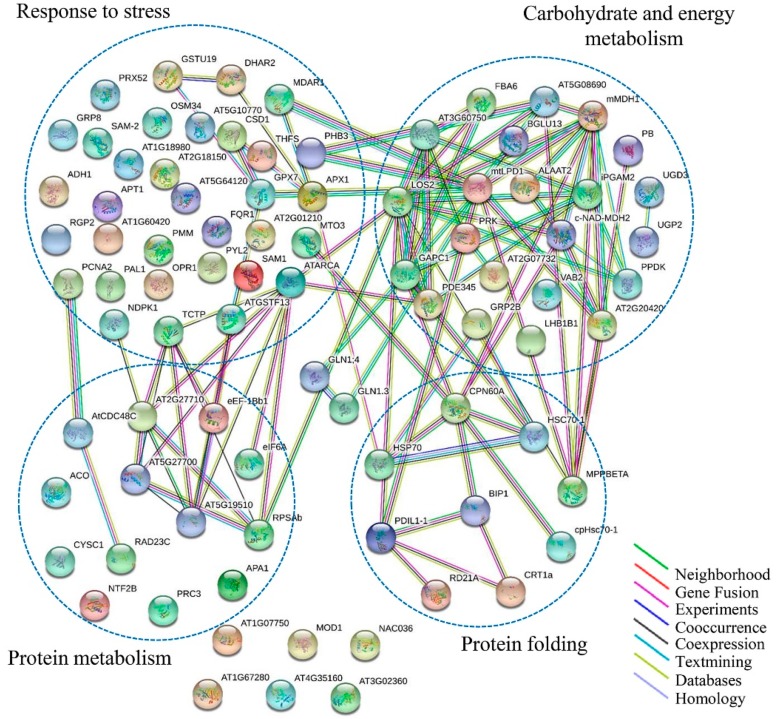
Analysis of protein interaction network by STRING 10.5. The Arabidopsis Information Resource (TAIR) homologous proteins from identified protein species were mapped by searching the STRING 10.5 software with a confidence level of 0.67. The colored lines between the proteins indicate the various types of interaction evidence.

The PPI network of all 138 DAPs was constructed using on line STRING 10.5 Software. All 138 DAPs were blasted against the *Arabidopsis thaliana* proteins database (TAIR 10; Table S7). DAPs were functional clusters according to the biological processes in which they are involved. STRING analysis revealed the functional links between DAPs in which the protein species involved in carbohydrate and energy metabolism, response to stress, protein metabolism, and protein folding processes were major clusters (Figure 7). Actually, these four clusters were not separated and together they formed a related-network in response to drought stress. The carbohydrate and energy metabolism, response to stress, and protein metabolism process groups contained more members, with interaction being concentrated on LOS2, ATARCA, and RPSAb, respectively. With respect to carbohydrate and energy metabolism, response to stress, and protein folding processes, CPN60A and HSP 70 were the most important nodes. Abbreviations of the specific protein species names in the network are shown in Table S7.

**Figure 8:**
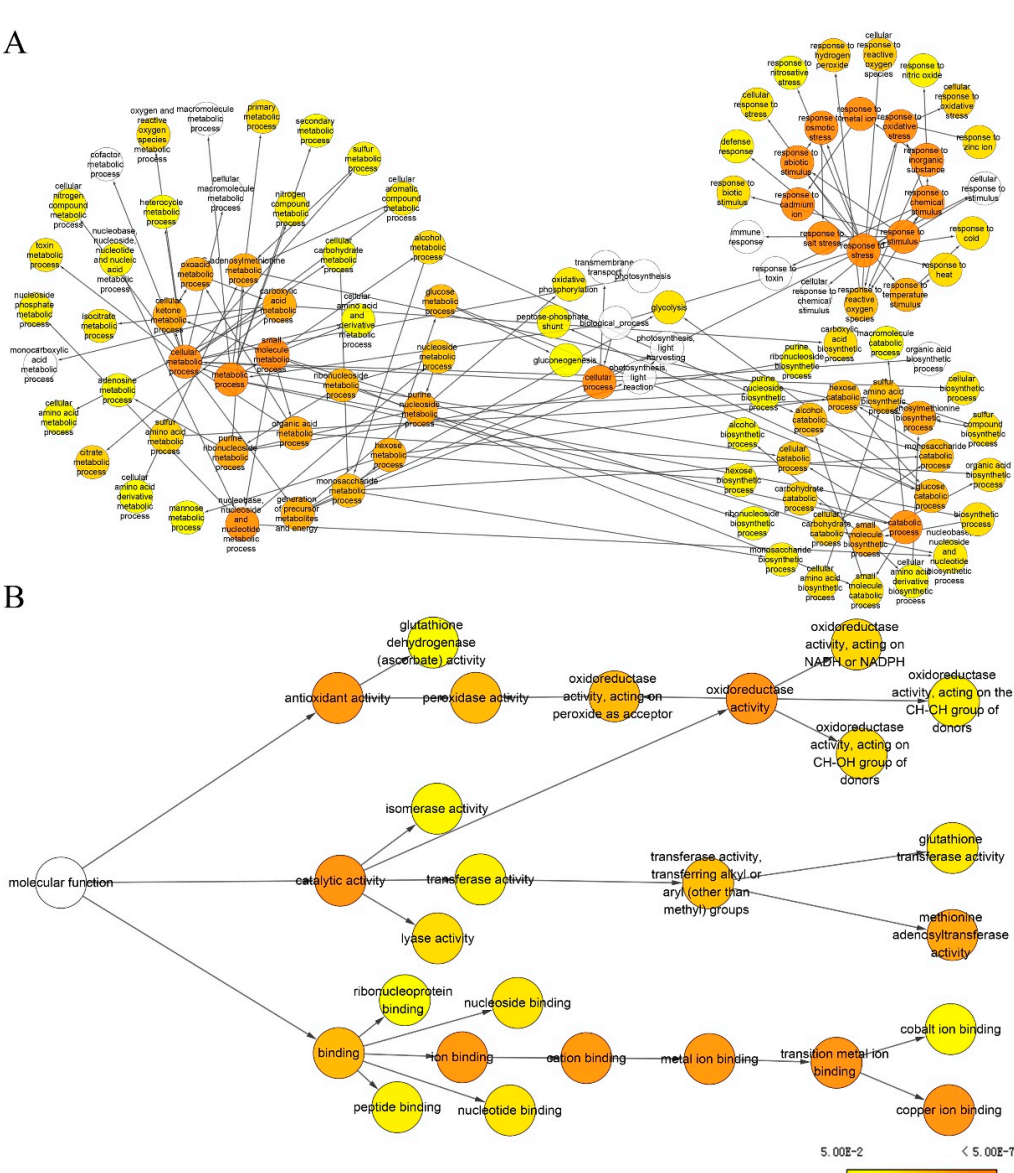
Biological pathway (**A**) and molecular function (**B**) networks generated by BiNGO. Homologous proteins were used for the gene ontology (GO) analysis. The size of the node is related to the number of proteins and the color represents the *p*-value for statistical significance of the overrepresented GO term (see the color scale on the right bottom).

To obtain statistically over- or under-represented categories of biological pathways and molecular functions related to drought treatment, BiNGO was used to analyze identified differential protein species (Figure 8, Tables S8 and S9). The results revealed that several overrepresented biological pathways were mostly significant (Figure 8A, Table S8), including response to metal ion (*p* =1.06 × 10^−28^), response to inorganic substance (*p* = 9.37 × 10^−28^), response to cadmium ion (*p* = 2.24 × 10^−26^), response to stimulus (*p* = 1.70 × 10^−22^), response to stress (*p* = 3.35 × 10^−21^), and response to chemical stimulus (*p* = 3.06 × 10^−20^). More specifically, metabolic processes (*p* = 2.99 × 10^−11^), small molecule metabolic process (*p* = 7.46 × 10^−8^), cellular metabolic processes (*p* = 7.60 × 10^−8^), and the S-adenosylmethionine metabolic process (*p* = 8.63 × 10^−6^), were significantly overrepresented. Meanwhile, a complete list of the enriched Gene Ontology (GO) molecular functions for the proteins is presented in Figure 8B and Table S9. Of them, several of the most highly enriched molecular functions included copper ion binding (*p* = 1.27 × 10^−16^), catalytic activity (*p* = 3.69 × 10^−11^), oxidoreductase activity (*p* = 1.14 × 10^−7^), methionine adenosyltransferase activity (*p* = 1.20 × 10^−7^), antioxidant activity (*p* = 1.81 × 10^−7^), transition metal ion binding (*p* = 8.68 × 10^−7^), ion binding (*p* = 1.91 × 10^−6^), and cation binding (*p* = 1.91 × 10^−6^).

## 3. Discussion

### 3.1. Morphological and Physiological Response of Wheat Seedlings to Exogenous WV Pretreatment

The effect of the exogenous WV seed soaking treatment on growth and stress tolerance of plants depended on the use of an optimum concentration because WV applied beyond a certain range might be detrimental. Our results indicated that soaking seeds with 1:900 WV gave optimal promotion to wheat seedlings.

ABA is a stress phytohormone that often accumulates in plants exposed to abiotic and biotic stress [27,28,29,30,31]. ABA is involved in defense priming in plants [28] and can activate antioxidative defense systems that contribute to the alleviation of stress [29,32]. Moreover, elevated ABA levels in plants can trigger signaling cascades downstream of phytohormones, such as salicylic acid (SA), which may also mitigate oxidative stress [30,31,33,34,35,36]. In our research, ABA content of shoots and roots were increased significantly in the WV treated group over those of the control group under both drought or non-drought stress. Our results indicated that WV can induce ABA biosynthesis in wheat seedlings. On the one hand, accumulation of ABA can regulate the stomatal apertures of leaves to prevent water loss; on the other hand, it can activate downstream antioxidative gene expression of shoots and roots to better resist subsequent drought stress.

Exogenous low concentrations of WV can cause slight oxidative stress in wheat roots but not oxidative damage. Our results showed that O^2−^ and H_2_O_2_ contents were higher in the WV treated group before drought stress than in the control group, whereas the content of MDA showed no significant difference between the two groups before drought stress. Moreover, the contents of O^2−^, H_2_O_2_, and MDA were significantly lower in the WV treated group than the control group after drought stress. Meanwhile, the activities of antioxidant enzymes were higher and related antioxidant genes were upregulated in the WV treated group under both drought and non-drought stress, which enabled them to cope better with continuous ROS production. This result indicated that a low concentration of WV acts as a stressor, having a slight effect on the oxidative status of the plant similar to that of stress-acclimating processes.

### 3.2. Protein Species Involved in Carbohydrate Metabolism and Energy Production

Carbohydrate metabolism regulates sugar synthesis and transformation as well as carbon partitioning, while drought stress disrupts carbohydrate metabolism in plants. In this study, a large proportion of the protein species whose abundance changed significantly under drought stress are associated with carbohydrate metabolism and energy production processes. Triosephosphate isomerase (TPI; spot 101, 104, 105), glyceraldehyde-3-phosphate dehydrogenase (GAPDH; spot 83, 84, 85), enolase (spot 37), and fructose bisphosphate aldolase (FBA; spot 72, 73, 74), were present at higher levels in the WV treated group over the control group. In plants, TPI are located in cytosol and chloroplast and are involved in several metabolic pathways, including glycolysis, gluconeogenesis, and Calvin cycle [37]. GAPDH, as a moonlighting protein, is involved in glycolysis and the Calvin cycle, but also played a vital role in redox signal transduction in plants [38,39]. In higher plants, FBA is located in cytosol and plastids, functioning in the Calvin cycle, glycolysis and gluconeogenesis. Previous studies showed that FBA could be redox-modified by glutathione (GSH) and participated in redox regulatory of *Arabidopsis thaliana* [40]. In our study, 3, 2, and 3 DAPs were identified as TPI, NAPDH, and FBA, respectively. They were considered as different protein species and were involved in different metabolic pathways. During drought stress in plants, the increased abundance of TPI, GAPDH, FBA and enolase could be related to the cellular requirement for extra energy in order to deal with stress and repair damage [41]. Our study indicated that WV can promote the wheat glycolysis metabolic pathway to produce more energy under drought stress. The study showed that the TCA cycle may be fueled by products derived from the degradation of protein and other macromolecules, in order to produce sufficient ATP to meet energetic demands under stress [42]. Aconitate hydratase (spot 6, 7) and malate dehydrogenase (spot 86) are components of the TCA cycle, an important source of energy for cells, and were present at higher levels in the WV treated group under drought stress. Aconitase isoforms are located in the mitochondria and cytosol [43]. Another role of aconitase is a “circuit breaker” that reduced electron flow through the mitochondrial electron transport chain and to a subsequent decrease of ROS [44]. In our study, this result indicated that WV can enhance TCA cycle speed in wheat roots to enable them to cope with subsequent drought stress. ATP synthase is the universal enzyme that manufactures ATP from ADP and provides energy for a large number of fundamental biological processes [13]. In the present study, the proteins related to ATP production (spot 35, 36, 38, 127) were found to be increased in abundance in the WV treated group under drought stress. During biotic and abiotic stress in plants, energy costs are high during stress acclimation, for example, the increased relative abundance of components of ATP-synthase [45]. Here, as a whole, the abundance of different subunits of ATP synthesis was increased in the WV treated group compared to the control plants; this change protected multiple normal metabolic processes dependent on ATP under drought stress. Our study suggested that the WV pretreatment regulated carbohydrate metabolism and, under drought stress, further enhanced carbohydrate synthesis and ATP production in wheat roots.

### 3.3. Protein Species Involved in the Stress Response

In the present study, O^2−^, H_2_O_2_ and MDA, which have the potential to cause peroxide damage and membrane lipid peroxidation. In general, within a certain threshold of abiotic stress, plants have a series of protective mechanisms to scavenge or reduce ROS and MDA levels and maintain the stability of cellular homeostasis [46]. These protective mechanisms include the activity of antioxidative proteins. In our study, protein species involved in the oxidative stress response were also identified; some anti-stress protein species, such as peroxidase 8 (spot 39, 40), peroxidase 1 (spot 49), pox1 (spot 78, 94), peroxidase 70 (spot 96), L-ascorbate peroxidase 1 (spot 99, 108, 110, 111, 112), glutathione transferase (spot 103, 113, 114), superoxide dismutase (spot 130), monodehydroascorbate reductase (spot 47), and dehydroascorbate reductase (spot 109) were more abundant in roots of the WV group than in control plants. Real-time PCR results of related antioxidative proteins (spot 47, 49, 56, 108, 114, 130), showed the same changing trend as the abundance of these protein species in both groups (Figure 4B). In plants, peroxidase was a protein superfamily and involved in countering effects of stress through signal transduction, strengthening of the cell wall [47], as well as the scavenging of toxic peroxides and ROS, accumulated under oxidative stresses [48]. Studies have indicated that peroxidase abundance and activity of peroxidase increased significantly in soybean roots under drought stress [49]. In plants, SOD is highly efficient at eliminating O^2-^, which can convert O^2−^ to molecular oxygen and H_2_O_2_. Subsequently, H_2_O_2_ is reduced to H_2_O by peroxidase [50]. Ascorbate peroxidase is one of the most important components for scavenging H_2_O_2_ [51]. GSH can combine with glutathione and a wide variety of hydrophobic and electrophilic compounds to eliminate cytotoxic compounds [52]. Studies have shown that GSTs were upregulated significantly in drought stressed wheat [53]. In our study, the contents of ROS and MDA were significantly increased in roots of the control and WV-treated groups under drought stress. However, the contents of ROS and MDA accumulated at a lower level in the WV-treated roots compared with the control group. There is no doubt that increased abundance of these anti-stress proteins restrained the accumulation of ROS and lowered damage induced by MDA in the WV treated group. These results suggest that WV pretreatment enhanced the antioxidant defense system to decrease oxidative damage under drought stress and provided a favorable environment for growth and development.

In addition to the above described DAPs involved in the stress response, *S*-adenosylmethionine synthetase is a member of the stress-induced family of genes [54]. Previous studies indicate that overexpression of S-adenosyl-l-methionine synthetase increase tomato plant tolerance to alkali stress through polyamine and hydrogen peroxide cross-linked networks [55]. In our study, four DAPs were identified as S-adenosyl-methionine synthase (spot 52, spot 53, spot 54, spot 106); we, therefore, suggest that its greater abundance in the WV treated group enhanced the capacity of plants to resist drought.

### 3.4. Protein Metabolism-Related Proteins

Protein synthesis, assembling, folding, and degradation are the main biologic process of protein metabolism [56]. In the present study, 24 DAP spots were involved in protein metabolism and were grouped into three functional subgroups: Proteins involved in protein synthesis, folding and degradation. In the first subgroup, elongation factor 1-delta (EF1D) and elongation factor 1-beta (EF1B; spot 87, spot 88) had a higher accumulation in the control group than the WV treated group. Elongation factors are proteins that play a central role in the elongation phase of protein synthesis in plants. Spots 133 and 138 were identified as 40S ribosomal protein and 60S acidic ribosomal protein, respectively, and their abundance was greater in the control group than in the WV treated group. 40S ribosomal protein and 60S acidic ribosomal protein are components of the ribosome machinery and are required for protein synthesis [57]. In our case, proteins suffered damage due to accumulated ROS in the control group root, increased abundance of the elongation factor and the related ribosomal protein could have caused the accumulated synthesis of proteins and replaced damaged proteins caused by peroxidation under drought stress in the control group plants. In contrast, ROS was eliminated over time by antioxidative proteins and this protected the stability of proteins in the roots of the WV treated group.

In the second subgroup, 3 DAPs (spot 8, spot 14, spot 15) were identified as 70 kDa heat shock proteins (HSPs), whose abundance was increased in roots of the WV treated group (Figure 3, Supplementary Tables S2 and S3). HSPs play crucial roles in protecting plants against stress and they are involved in a wide range of crucial cellular processes [58]. Previous studies indicate that HSP 70 can prevent the aggregation of denatured proteins and assist in the refolding of nonnative proteins caused by environmental stress [59]. Our results indicated that the accumulation of ROS caused instability of proteins under drought treatments. An increased abundance of HSPs in roots of the WV treated group provided a more effective protective mechanism in response to oxidative stress.

In the third subgroup, 3 DAPs (spot 79, spot 80, spot 89) were identified as aspartic proteinase proteins (Table S5). Aspartic proteinase is an endopeptidase and is active under acidic pH conditions [60]. Previous studies show that *SPAP1*, which encodes a typical aspartic protease protein, is responsible for leaf senescence in the sweet potato [60]. Many studies indicate that aspartic proteinase participates in the PCD process of many plant organs [61,62,63,64]. In our case, excessive ROS was not effectively removed from roots in the control group, which resulted in the accumulation of dysfunctional amounts of proteins. An increased abundance of aspartic proteinase in the control group could have effectively hydrolyzed dysfunctional proteins; moreover, aspartic proteinase will have promoted apoptosis of damaged cells by participating in the PCD process. Spot 102 was identified as a proteasome subunit, which controlled the protein degradation process. A previous study indicated that the ubiquitin-proteasome system (UPS) plays an important role in response to environmental stress such as drought, salinity, cold, and nutrient deprivation. Moreover, UPS has shown to be related to the production of ABA and participate in signal transduction pathway [65]. In our case, the abundance of the proteasome subunit was increased in roots of the control group. This indicated that excessive ROS induced oxidative damage to protein structure and function and these dysfunctional proteins needed to be degraded immediately in roots of the control group to maintain the stability of the normal mechanical processes of cellular homeostasis.

### 3.5. Proteins Involved in Secondary Metabolism

Jasmonic acid (JA) and salicylic acid (SA) are important secondary metabolites in plants, being involved in the various metabolic process, particularly in response to biotic and abiotic stress in plants [66,67]. Previous studies indicate that JA accumulates rapidly after biotic and abiotic stressors [67,68], that trigger the biosynthesis of JA from linolenic acid, suggesting that JA is an important stress-signaling molecule in plants. SA has also been identified as an endogenous regulatory signal in plants, particularly during plant defense against pathogens and drought stress [13,66,69]. In addition, pretreatment with low concentration SA significantly enhances the growth of wheat seedlings and the shoots and roots of soybean [13,70]. In our case, 4 DAPs (spot 64, spot 67, spot 68, spot 69) were identified as 12-oxophytodienoate reductase (OPR), which is a key enzyme in JA biosynthesis, and their abundance was higher in the roots of the WV treated group compared with the control. The expression pattern analysis of *TaOPR* showed the same results as for protein quantification (Figure S8). Previous studies indicate that wounded plants rapidly accumulate JA, and this signal activates the expression of early response genes [71]. In the WV treated group, increased accumulation of OPR may have accelerated the biosynthesis of JA; accumulated JA triggers expression of defense genes via the octadecanoid pathway or by acting directly on the genes. When wheat plants suffered drought stress, a faster and effective response triggered by accumulated JA was initiated in WV-treated roots. In our study, spot 12 and spot 13 were identified as phenylalanine ammonia-lyase (PAL), which plays a significant role in the biosynthesis of SA. A previous study indicates that PAL activity and the content of SA in pharbitis were both up-regulated under the stress treatment [72]. In our case, an increase of PAL in the WV treated group promoted the biosynthesis of SA. Moreover, the q-PCR result of *TaPAL* showed up-regulated expression in the WV treated group under drought stress and non-stress conditions (Figure S8). SA activates various genes that encode antioxidants, chaperones, and heat shock proteins to resist drought stress.

### 3.6. WV can Initiate An Early Defense Mechanism to Mitigate Subsequent Drought Stress

**Figure 9:**
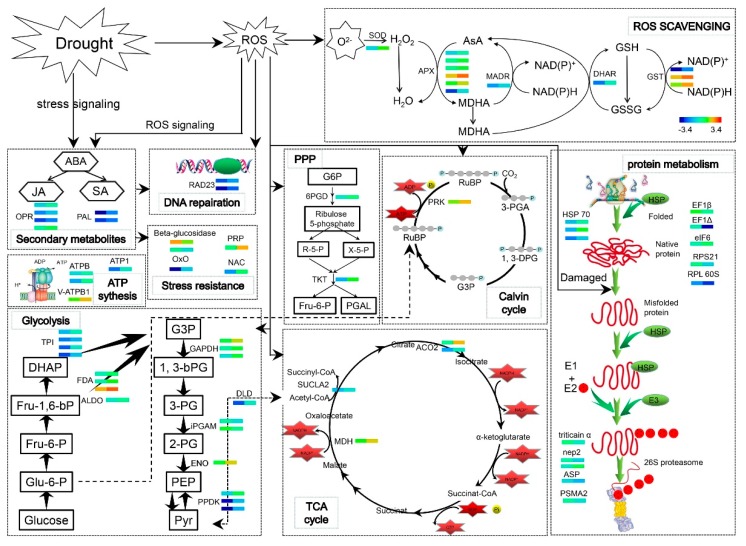
Diagram of the main metabolic pathways regulated by WV under drought stress. Each small colored square represents an individual protein under different treatments (from left to right, control and WV treated groups, respectively). Relative levels of expression are shown by a color gradient from low (blue) to high (red). SOD: Superoxide dismutase; APX: L-ascorbate peroxidase; MADR: Monodehydroascorbate reductase; DHAR: dehydroascorbate reductase; GST: glutathione transferase; OPR: 12-oxophytodienoate reductase; PAL: Phenylalanine ammonia-lyase; RAD23: DNA repair protein RAD23; ATPB: ATP synthase beta subunit; V-ATPB1: Vacuolar ATPase subunit B1; Oxo: Oxalate oxidase GF-2.8; PRP: Pathogenesis-related protein; NAC: NAC transcription factor; TPI: Triosephosphate isomerase; FDA: Fructose-bisphosphate aldolase cytoplasmic isozyme; ALDO: Aldolase; GAPDH: Lyceraldehyde-3-phosphate dehydrogenase; iPGAM: 2,3-bisphosphoglycerate-independent phosphoglycerate mutase; ENO: Enolase; PPDK: Phosphate dikinase 1; DLD: Dihydrolipoyl dehydrogenase 1; SUCLA2: Succinyl-CoA ligase [ADP-forming] subunit beta; MDH: Malate dehydrogenase 1; ACO2: Aconitate hydratase; 6PGD: 6-phosphogluconate dehydrogenase; TKT: Transketolase; PRK: Phosphoribulokinase; EF1β: Elongation factor 1-beta; EF1Δ: Elongation factor 1-delta; eIF6: Eukaryotic translation initiation factor 6; RPS21: 40S ribosomal protein S21; RPL 60S: 60s acidic ribosomal protein-like protein; nep2: Aspartic proteinase nepenthesin-2; ASP: Aspartic proteinase; PSMA2: Proteasome subunit alpha type-2.

Our results showed ABA levels were significantly increased in the shoots and roots of the WV treated group. ABA accumulated in the shoots and roots of the WV treated group to rapidly regulate stomatal aperture and the expression of defense-related genes when the wheat plants underwent drought stress, thus conferring resistance to drought stress. Meanwhile, comparative proteomic analysis revealed that WV promoted the biosynthesis of JA and SA, which regulated downstream related anti-stress gene expression with ABA through signal transduction. In our present work, soaking with WV launched an early defense mechanism before drought stress began. Soaking with WV induced the production of ROS, which remained within a safe threshold because of increased activities of antioxidant enzymes and the effective opening of the defense system. During drought stress, ROS content was significantly higher in the roots of the control group and they suffered oxidative damage. Comparative proteomic analysis revealed that carbohydrate metabolism was inhibited, and this accelerated the degradation of damaged proteins in the control roots under drought stress condition. However, proteomic analysis and results of the determination of physiological indices indicated that ROS was effectively removed by the increased abundance of antioxidative and related stress proteins in WV pretreated roots after drought stress. An overview of the main metabolic pathways regulated by WV under drought stress is shown in Figure 9. These results indicated that WV can promote the growth of wheat shoots and roots, and also improve their tolerance to drought stress.

## 4. Materials and Methods

### 4.1. Plant Materials

Wheat (*Triticum aestivum* L.) cultivar “Zhoumai 18” seeds were sterilized with 70% ethanol and 10% NaClO followed by a thorough washing with sterilized water. Seeds were then soaked in sterilized water supplemented with various volumes of primary WV (Yixin Bio-energy Technology Development Co. LTD, Yangling, China; composition of primary WV is listed in Table S1) for 3 days. The seeds were grown in a greenhouse under a day/night temperature regime of 25 °C, under 12 h d^−1^ illumination, light intensity of 300 μmol m^−2^·s^−1^ and a relative humidity of 60%–70%. The experimental design was presented in Figure S1.

To explore the optimal various concentrations of WV on wheat seedling growth, 25 sterilized seeds were soaked in sterilized water supplemented with control 0 (control group), 1:300, 1:600, 1:900, 1:1200, and 1:1500 various volumes of WV [primary WV:ddH_2_O_2_ (V:V); WV treated groups], respectively, for 3 days. The experiments were laid out in a completely randomized design (CRD). Three biological replications (5 wheat plants) were set for each treatment. Then soaking seeds were distributed in a 115 × 115 mm sterile germination box with two layers of filter paper saturated with 10 mL of sterilized water. Seeds were dampened with 5 mL of water every day, for one week. The aerial parts (shoots) and roots of both the control and WV treated groups (1:300, 1:600, 1:900, 1:1200, 1:1500) from day 2 to day 6 after seed germination were collected for fresh weight (FW) and dry weight (DW) analysis.

In order to explore the effects of WV treatments on the drought tolerance of wheat, 50 sterilized seeds were soaked in sterilized water supplemented with control 0 (control group) and 1:900 primary WV [primary WV:ddH_2_O_2_ (V:V); WV treated groups], respectively, for 3 days. Three biological replications (50 wheat plants) were set for each treatment. Subsequently soaked seeds were distributed in a 115 × 115 mm sterile germination box with two layers of filter paper saturated with 10 mL of sterilized water, for 4 days. On the fifth day after germination, seedlings of both the control and WV treated groups (1:900) were transferred to a Hoagland nutrient solution with −1 MPa PEG 6000 (simulated drought stress), respectively, for 2 days. Thereafter, shoots and roots were collected from the control and WV treated groups (1:900) from day 3 to day 6 to determine the abscisic acid (ABA) content. Roots were then collected from the control and WV treated groups (1:900) from day 3 to day 6 to determine O^2−^, H_2_O_2_, and malonaldehyde (MDA) content, and the generation rate and the activities of superoxide dismutase (SOD, EC.1.15.1.1), catalase (CAT, EC 1.11.1.6), and guaiacol peroxidase (POD, EC1.11.1.7). For proteomics, roots from both the control and WV treated groups (1:900) were collected on the sixth day (under drought stress treatment conditions for 2 days). For real-time PCR analysis, shoots and roots were collected from the control and WV treated groups (1:900) from day 3 to day 6.

### 4.2. Determination of O^2−^ Formation Rate and H_2_O_2_ Content

Determination of O^2−^ and the H_2_O_2_ content were performed according to Song et al. [73], with minor modifications: Roots were ground to powder in liquid nitrogen; centrifugal force was 7000× *g*. Briefly, the reaction was initiated in assay solution (65 pH 7.8 mM phosphate buffer, 10 mM hydroxylamine chlorhydrate, 17 mM sulfanilamide and 7 mM α-naphthylamine). Absorbance at 530 nm was measured and the formation rate of O^2−^ was calculated from a standard curve of NaNO_2_. Determination of H_2_O_2_ content was performed according to Song et al. [73].

### 4.3. Determination of Antioxidant Enzyme Activity

The activity of SOD, CAT, and POD was determined according to Song et al. [73]. To extract antioxidant enzymes, 0.5 g of fresh roots were ground in liquid nitrogen, then root powder was transferred to a 50 mM cool phosphate buffer [containing 1% (*w*/*v*) polyvinylpyrrolidone, pH 7.0] and centrifuged at 4 °C and 15,000× *g* for 20 min. The supernatant was used for enzyme activity assays.

For the estimation of SOD activity, the reaction was initiated in an activity assay solution (50 μM NBT, 1.3 μM riboflavin, 13 mM methionine, 75 nM EDTA, 50 mM pH 7.8 phosphate buffer, and enzyme extract). The absorbance at 560 nm was determined with a spectrophotometer. For the measurement of CAT activity, the reaction was initiated in the activity assay solution (50 mM pH 7.8 phosphate buffer, 15 mM H_2_O_2_, and enzyme extract). The decrease in absorbance of activity assay solution at 240 nm was read every 20 s. For the determination of POD activity, the reaction was initiated in the activity assay solution (50 mM pH 5.0 sodium acetate buffer, 20 mM guaiacol, 40 mM H_2_O_2_, and enzyme extract). The increase in absorbance of activity assay solution at 470 nm was recorded every 20 s.

### 4.4. Determination of MDA Content

MDA content was estimated according to Song et al. [73]. Briefly, roots (0.5 g) were homogenized in 20% (*v*/*v*) TCA and 0.5 (*v*/*v*) thiobarbituric acid (TBA). The supernatants after centrifugation were incubated at 95 °C for 10 min and cooled in ice immediately. The absorbance at 532 nm was read.

### 4.5. Quantitative Determination of ABA Content

The plant hormone ABA was extracted according to the method described by Shi et al. [74], with minor modifications. Fresh root samples (ca. 1 g) were ground in liquid nitrogen, then powder was suspended in 10 mL of 80% (*v*/*v*) methanol containing 200 mg·L^−1^ of butylated hydroxytoluene and 500 mg·L^−1^ of citric acid monohydrate on ice. The mixture was subsequently shaken overnight at 4 °C before centrifugation for 30 min at 8000× *g* and 4 °C. The supernatant was collected. The precipitate was extracted twice for 2 h, the supernatants were combined and subsequently dried under N_2_ and resuspended in 500 μL of 80% methanol. The phytohormone concentrations in the extracts were analyzed using an LC-20AT high performance liquid chromatography system (Shimadzu, KinhDo, Japan) and an API 2000™ electrospray tandem mass spectrometer (AB Sciex, Foster City, CA, USA). Two microliter samples were separated within a Wondasil™ C18 column (5 μm, 4.6 × 150 mm; Shimadzu). ABA ((±)-ABA, A1049; Sigma, St. Louis, MO, USA) was used to prepare standard curves for the determination of hormone concentrations in samples.

### 4.6. Protein Extraction

Protein extraction was performed as described by Valledor et al. [75], with minor modifications. Wheat roots were ground to a fine powder with liquid nitrogen. The ground root powder was homogenized with −20 °C ice-cold extraction buffer [10% (*w*/*v*) TCA, 0.07% β-mercaptoethanol (β-ME; *v*/*v*), and 1mM PMSF], then proteins were precipitated overnight. The following day, the mixture was centrifuged at 20,000× *g* for 30 min and the pellet was resuspended in 2 mL of −20 °C ice-cold acetone [0.07% β-mercaptoethanol (β-ME; *v*/*v*), and 1 mM PMSF]; this was repeated 3 times. Finally, the pellet was collected, lyophilized with vacuum freeze-drying equipment, and stored at −80 °C.

### 4.7. 2-DE and Gel Image Analysis

2D-PAGE was performed according to the method described by Valledor et al. [75], with minor modifications. The root proteins were solubilized in lysis solution and proteins concentration was determined using Bio-Rad Protein Assay Kit II (Bio-Rad, Shanghai, China), with Bovine serum albumin (BSA) as a standard protein. About 900 μg of protein was separated on a 17 cm pH 4–7 linear IPG strip (Bio-Rad) and actively rehydrated at 50 V for 14 h at 20 °C. Subsequently, focusing was performed under following conditions: 250 V for 1 h, 500 V for 1 h, 1000 V for 1 h, 8000 V for 4 h, and 8000 V to achieve 80,000 V-h. Strips were immediately equilibrated twices. The Second-dimension electrophoresis was performed on 12% polyacrylamide gels. Gels were stained with Coomassie Brilliant Blue (CBB) G-250. Each sample was run in 3 independent biological replicates.

Gels were visualized using a GS-900 Calibrated Densitometer (Bio-Rad, Taiwan, China) at a resolution of 600 dpi. Images were analyzed using the analytical software PDQuest 2-DE 8.0.1 (Bio-Rad, Hercules, CA, USA) for spot detection, gel matching, and statistical analysis of spots. The selection of protein spots of interest for analysis by MS was based on a fold change ≥1.5 (*p* < 0.05).

### 4.8. In-gel Digestion and MALDI-TOF/TOF MS Analysis

The DAP spots were excised, washed, de-stained, and dehydrated. Subsequently, protein spots were digested with trypsin. The supernatant was collected, and the resultant peptides were extracted twice with 0.1% trifluoroacetic acid (TFA) and 60% ACN. Then, the supernatants were combined. Mass spectra were collected using a 5800 MALDI Time of Flight (TOF)/TOF^TM^ analyzer (AB Sciex, Foster City, CA, USA) and analyzed using TOF/TOF^TM^ Series Explorer^TM^ Software V4.1.0 (AB Sciex, Redwood City, CA, USA).

MS/MS mass spectra data were searched against the NCBInr databases with a taxonomy parameter set to *Viridiplantae* using the Mascot search engine. The search parameters were set as follows: One missed cleavage, peptide tolerance set to 100 ppm, MS/MS tolerance of 0.5 Da, peptide charge of 1+, carbamidomethylation and oxidation of methionines allowed as fixed modification variable modification.

### 4.9. Total RNA Isolation and Real-Time PCR

Total RNA was extracted from wheat shoots and roots of the control and WV treated groups using an OMEGA plant RNA kit (R6827, Omega Bio-tek, Norcross, GA, USA), and cDNA was reverse transcribed from 1 μg of total RNA using the GoScript™ Reverse Transcription System (A5001, Promega, Madison, WI, USA). Relative quantification of gene expression by qPCR was performed on a QuantStudio 3 Real-Time PCR System (Thermo Fisher Scientific, Singapore, Singapore). The primers used for qPCR were designed using the qPrimerDB database [76], Oligo 7 and Beacon Designer™ 8.0 software. The sequence of the primers can be found in the Supplementary Table S3. Wheat actin gene was used as the endogenous control which remained stable throughout the drought treatment [77,78]. qPCR was performed in an optical 96-well plate, including 10 μL 2 × GoTaq^®^ qPCR Master Mix (A6002, Promega, Madison, WI, USA), 2 μL 1:5-diluted template cDNA, and 0.2 μM of each gene-specific primer, in a final volume of 20 μL, using the following thermal cycles: 95 °C for 1 min, 40 cycles of 95 °C for 10 s, 60 °C for 1 min. Disassociation curve analysis was performed as follows: 95 °C for 15 s, 60 °C for 1min, and 95 °C for 15 min. Relative expression levels were calculated by the 2^−ΔΔ*C*t^ method [79].

### 4.10. Bioinformatic Analysis

The prediction of transmembrane domains (TMDs) of the identified DAPs was carried out using TMpred (http://www.ch.embnet.org/software/TMPRED_form.html). The grand average of hydropathicity (GRAVY) value for each DAP was calculated using the Protein GRAVY tool (http://www.bioinformatics.org/sms2/protein_gravy.html). Cellular locations of DAPs were performed through WoLF PSORT (https://wolfpsort.hgc.jp/) and (http://www.csbio.sjtu.edu.cn/bioinf/plant-multi/). All identified DAPs were blasted against the *Arabidopsis thaliana* TAIR 10 (The Arabidopsis Information Resource) protein database (http://www.arabidopsis.org/) for obtaining the annotated protein information to conduct a PPI network using the online analysis tool STRING 10.5. Biological processes and cellular component were predicted by the BiNGO plugin of Cytoscape software (version 3.6.0, San Diego, CA, USA).

### 4.11. Statistical Analysis

Principal component analysis (PCA) was performed [80,81] by SPSS software (version 22.0, IBM Corporation, Armonk, NY, USA) to identify homogeneous biological replicates and the difference between the control group and WV-treated group. In our study, coefficient and KMO and Bartlett’s test of sphericity were used for dimension reduction analysis. The volume of DAPs was estimated using the built-in statistical modules of PDQuest 8.01 by applying a log transformation and a *t*-test. The results were presented as mean ± standard deviation (SD) from three independent biological replicates. One-way analysis of variance (ANOVA) multiple comparisons was performed to calculate statistical significance; *p* < 0.05 was considered statistically significant. Graphical presentation of the data was performed using Originlab 2018b software (OriginLab Corporation, Northampton, MA, USA).

## 5. Conclusions

During wheat seedling growth, young seedlings are susceptible to water deficiency. However, a well-developed root system can improve wheat plants’ ability to defend against drought stress. Pretreatment soaking in appropriate concentrations of wood vinegar significantly promoted root and seedling growth. Moreover, WV was able to initiate an early defense mechanism to mitigate subsequent drought stress. In this process, ROS was effectively removed through the increased abundance of antioxidative and other related stress proteins. A battery of protective mechanisms in the WV soaked seed treatment helped to maintain the stability of the normal mechanical processes of cellular homeostasis and metabolism.

## Figures and Tables

**Table 1:** Effect of different concentrations of wood vinegar on the fresh weight (FW) and dry weight (DW) of shoots of five wheat seedlings.

Treatments		CK	1:300	1:600	1:900	1:1200	1:1500
2d	FW(mg)^1^	71.1 ± 1.75b	60.8 ± 1.87a	76.9 ± 1.5d	78.2 ± 1.6d	74.1 ± 1.43c	73.1 ± 1.25bc
	DW(mg)^1^	7.7 ± 0.35b	6.9 ± 0.11a	10.9 ± 0.49d	12.9 ± 0.61e	9.9 ± 0.51c	9.6 ± 0.25c
3d	FW(mg)^1^	116.6 ± 6.26b	90 ± 2.2a	127.6 ± 4.05de	134.7 ± 5.29e	126.5 ± 3.65cd	119.7 ± 1.89bc
	DW(mg)^1^	14.1 ± 0.53a	12.5 ± 0.6a	19.5 ± 1.35c	23.5 ± 1.12d	17.7 ± 0.76b	16.8 ± 0.64b
4d	FW(mg)^1^	179 ± 3.63b	152.9 ± 3.12a	189.4 ± 3.72c	203 ± 4.54d	192.3 ± 2c	188.7 ± 1.46c
	DW(mg)^1^	21.8 ± 0.67b	17.9 ± 0.66a	26.4 ± 0.38d	29.2 ± 0.5e	26.4 ± 0.65d	23.7 ± 0.23c
5d	FW(mg)^1^	249.7 ± 1.45b	211.8 ± 1.71a	272.1 ± 1.96d	286.9 ± 2.11e	270.7 ± 1.45d	262.7 ± 1.59c
	DW(mg)^1^	29.9 ± 0.64b	24.2 ± 0.32a	37.9 ± 0.75d	42.2 ± 0.2e	37.6 ± 0.35d	32 ± 0.21c
6d	FW(mg)^1^	312.3 ± 2.07b	302.9 ± 1.53a	340.3 ± 1.8d	363.7 ± 2.1e	335.3 ± 1.8c	332.8 ± 2.03c
	DW(g)^1^	37.7 ± 0.47b	35.4 ± 0.67a	48.3 ± 0.72d	54 ± 0.31e	45.7 ± 0.47c	38 ± 0.71b

^1^ Values are mean ± SD from three dependent experiments (biological replicates). Data with different letters in the same row indicate a significant difference at the *p* < 0.05 level.

**Table 2:** Effect of different concentrations of wood vinegar on the FW and DW of roots of five wheat seedlings.

Treatments		CK	1:300	1:600	1:900	1:1200	1:1500
2d	FW(mg)^1^	77.9 ± 1.4b	72 ± 1.96a	81.2 ± 1.31c	90.1 ± 1.42d	82.3 ± 1.25c	80.1 ± 1.18bc
	DW(mg)^1^	5.6 ± 0.35a	5.2 ± 0.3a	7.9 ± 0.35c	10.5 ± 0.2d	6.7 ± 0.25b	6.2 ± 0.21b
3d	FW(mg)^1^	91.5 ± 3.27ab	89.8 ± 2.39a	101.1 ± 3.72c	126.8 ± 3e	119.8 ± 4.58d	93.1 ± 2.69b
	DW(mg)^1^	6.3 ± 0.32a	6.8 ± 0.36a	10.1 ± 0.25c	18.7 ± 0.56d	10.4 ± 0.53c	7.7 ± 0.42b
4d	FW(mg)^1^	169.3 ± 1.65a	178 ± 1.61b	184.7 ± 1.55c	206.6 ± 1.67e	190.3 ± 1.05d	171 ± 1.15a
	DW(mg)^1^	14.3 ± 0.2a	15.5 ± 0.46b	19.9 ± 0.45c	27.4 ± 0.72e	23.9 ± 0.32d	15.7 ± 0.2b
5d	FW(mg)^1^	213.2 ± 2.05a	218.2 ± 2.11b	232.5 ± 1.56c	257.4 ± 1.22d	217.8 ± 1.76b	210.9 ± 2.27a
	DW(mg)^1^	17.8 ± 0.26a	21.7 ± 1.03b	26.5 ± 0.6c	33.7 ± 1.21d	26.7 ± 0.86c	18.1 ± 0.99a
6d	FW(mg)^1^	267.6 ± 2.12a	286.3 ± 2.03b	295.1 ± 4.41c	310.2 ± 1.65d	282.3 ± 2.04b	263.2 ± 1.78a
	DW(mg)^1^	21.4 ± 0.7a	27.9 ± 0.38b	35.2 ± 0.36d	41.5 ± 0.84e	33.7 ± 0.85c	21.6 ± 0.45a

^1^ Values are mean ± SD from three dependent experiments (biological replicates). Data with different letters in the same row indicate a significant difference at the *p* < 0.05 level.

## Supplementary Materials

Supplementary materials can be found at https://www.mdpi.com/1422-0067/20/4/943/s1. Figure S1. Experimental design used in the study; Figure S2. Supplementary pictures in the study; Figure S3. 2-DE image of wheat root proteomes in the control and WV-treated groups; Figure S4. Principal component analysis of the control and WV-treated groups; Figure S5. Master gel; Figure S6. Typical examples of DAPs showing different profiles; Figure S7. Hierarchical clustering of protein species of all 13 categories in both the control and WV treated groups; Figure S8. Expression pattern analysis of *TaOPR* and *TaPAL* genes in wheat roots in both the control and WV treated groups before and after the drought stress; Table S1. Chemical compounds identified in the WV; Table S1. Supplementary data in the study; Table S3. Specific primers used in this study; Table S4. Fold change (Ratio) of DAPs between the WV-treated and the control groups under drought stress; Table S5. MS/MS data, GRAVY, numbers of TMDs and subcellular localization of DAPs in wheat roots under drought stress; Table S6 Log-transformed values of each DAP in both the control and WV-treated groups; Table S7. DAPs blasted against the TAIR database; Table S8. Biological pathways generated for wheat roots; Table S9. Molecular functions generated for wheat roots. The mass spectrometry proteomics data have been deposited to the ProteomeXchange (http://proteomecentral.proteomexchange.org) Consortium via the PRIDE partner repository with the dataset identifier PXD012150.

## Abbreviations

2-DE	Two-dimensional gel electrophoresis
6PGD	6-phosphogluconate dehydrogenase
ABA	Abscisic acid
ACO2	Aconitate hydratase
ADH1A	Alcohol dehydrogenase
ALDO	Aldolase
APX1	L-ascorbate peroxidase 1
ASP	Aspartic proteinase
ATPB	ATP synthase beta subunit
CAT	Catalase
DEPs	Differential expression proteins
DHAR	Dehydroascorbate reductase
DLD	Dihydrolipoyl dehydrogenase 1
EF1β	Elongation factor 1-beta
EF1Δ	Elongation factor 1-delta
eIF6	Eukaryotic translation initiation factor 6
ENO	Enolase
FDA	Fructose-bisphosphate aldolase cytoplasmic isozyme
GAPDH	Glyceraldehyde-3-phosphate dehydrogenase
GRAVY	Grand average of hydropathicity value
GST	Glutathione transferase
iPGAM	2,3-bisphosphoglycerate-independent phosphoglycerate mutase
JA	Jasmonic acid
MDA	Malonaldehyde
MDAR	Monodehydroascorbate reductase
MDH	Malate dehydrogenase 1
Mr	Monoisotopic mass
NAC	NAC transcription factor
NECD	9-*cis*-epoxycarotenoid dioxygenase
nep2	Aspartic proteinase nepenthesin-2
OPR	12-oxophytodienoate reductase
Oxo	Oxalate oxidase
PA	Pyroligneous acid
PAL	Phenylalanine ammonia-lyase
pI	Isoelectric point
POD	Guaiacol peroxidase
POX1	Peroxidase 1
PPDK	Phosphate dikinase 1
PPI	Protein–protein interaction
PRK	Phosphoribulokinase
PRP	Pathogenesis-related protein
PSMA2	Proteasome subunit alpha type-2
q-PCR	Real-time PCR
RAD23	DNA repair protein RAD23
ROS	Reactive oxygen species
RPL 60S	60s acidic ribosomal protein-like protein
RPS21	40S ribosomal protein S21
RQ	Relative quantification
SA	Salicylic acid
SOD	Superoxide dismutase
SUCLA2	Succinyl-CoA ligase [ADP-forming] subunit beta
TKT	Transketolase
TMDs	Transmembrane domains
TPI	Triosephosphate isomerase
V-ATPB1	Vacuolar ATPase subunit B1
WV	Wood vinegar
