# Supplementary material for: Root Proteomics Reveals the Effects of Wood Vinegar on Wheat Growth and Subsequent Tolerance to Drought Stress
Source: Int J Mol Sci. 2019 Feb 21;20(4):943. doi: 10.3390/ijms20040943 (PMC6413028; doi:10.3390/ijms20040943)
Supplement: Supplementary file 1 [file ijms-20-00943-s001.zip › proofed version_ijms-449598-supplementary/Supplementary Figures.pptx]

## Slide 1
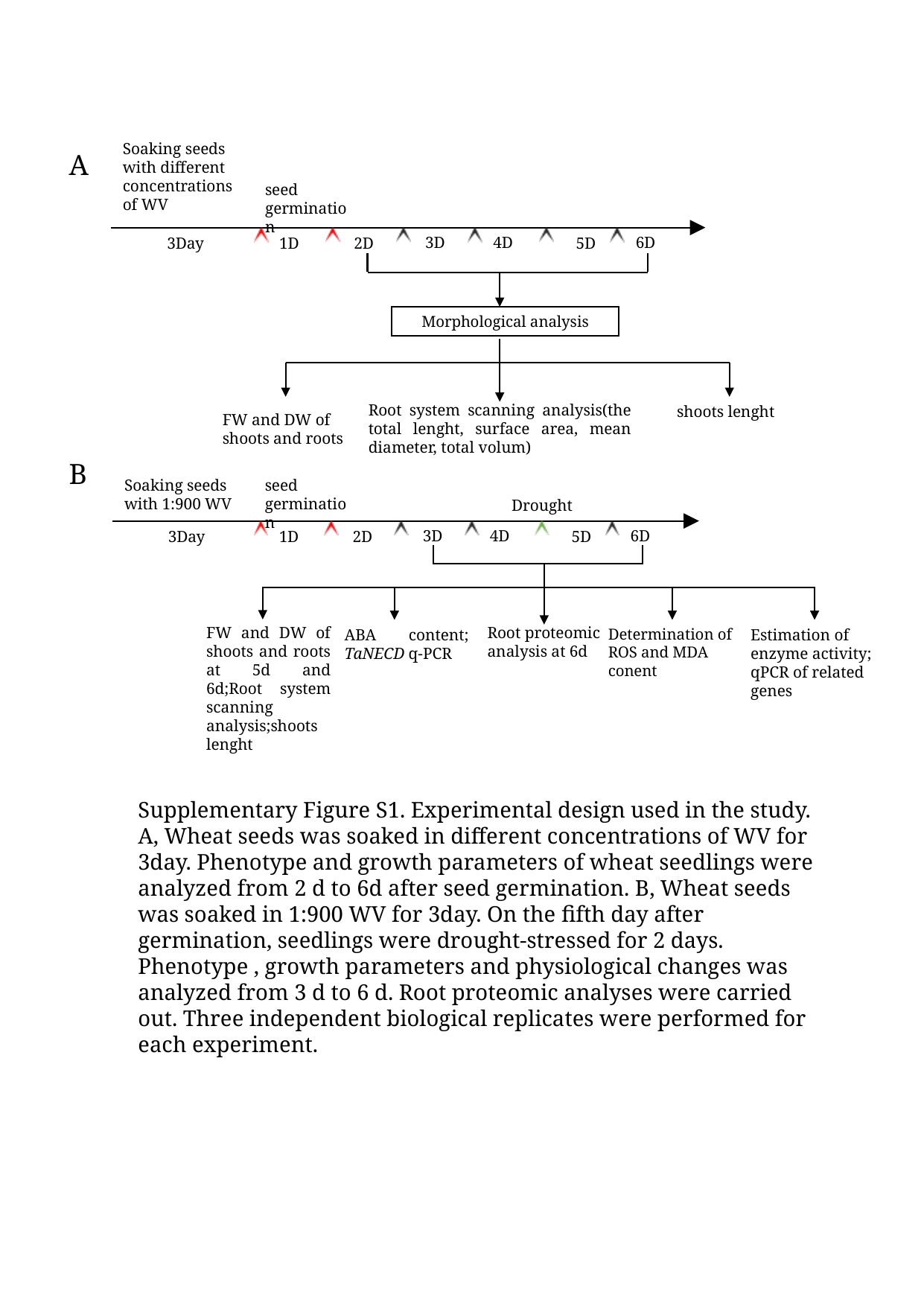

Soaking seeds with different concentrations of WV
A
seed
germination
3D
4D
6D
3Day
1D
2D
5D
Morphological analysis
Root system scanning analysis(the total lenght, surface area, mean diameter, total volum)
shoots lenght
FW and DW of shoots and roots
B
Soaking seeds with 1:900 WV
seed
germination
Drought
3D
4D
6D
3Day
1D
2D
5D
FW and DW of shoots and roots at 5d and 6d;Root system scanning analysis;shoots lenght
Root proteomic analysis at 6d
Determination of ROS and MDA conent
Estimation of enzyme activity; qPCR of related genes
ABA content; TaNECD q-PCR
Supplementary Figure S1. Experimental design used in the study. A, Wheat seeds was soaked in different concentrations of WV for 3day. Phenotype and growth parameters of wheat seedlings were analyzed from 2 d to 6d after seed germination. B, Wheat seeds was soaked in 1:900 WV for 3day. On the fifth day after germination, seedlings were drought-stressed for 2 days. Phenotype , growth parameters and physiological changes was analyzed from 3 d to 6 d. Root proteomic analyses were carried out. Three independent biological replicates were performed for each experiment.

## Slide 2
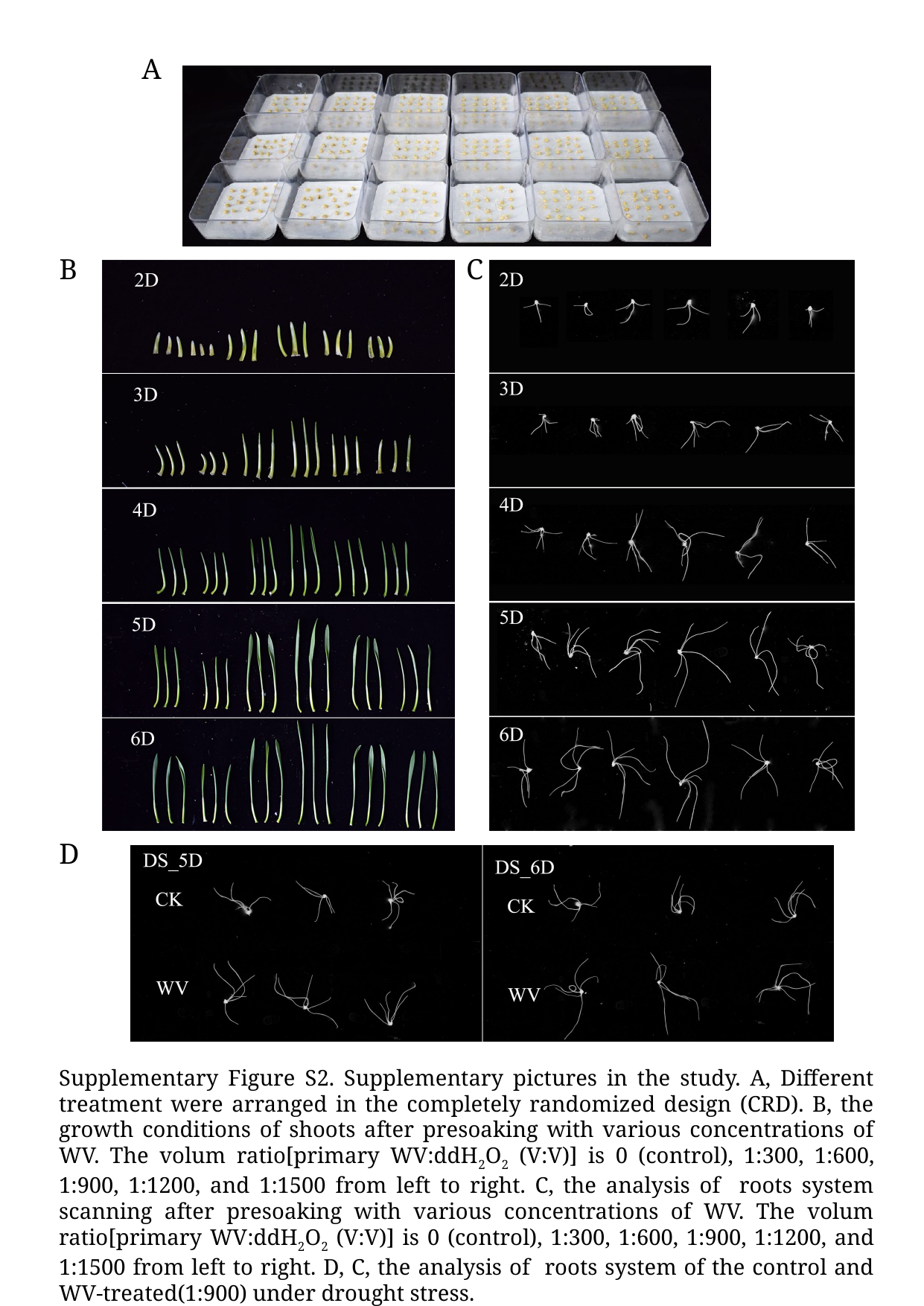

A
B
C
D
Supplementary Figure S2. Supplementary pictures in the study. A, Different treatment were arranged in the completely randomized design (CRD). B, the growth conditions of shoots after presoaking with various concentrations of WV. The volum ratio[primary WV:ddH2O2 (V:V)] is 0 (control), 1:300, 1:600, 1:900, 1:1200, and 1:1500 from left to right. C, the analysis of roots system scanning after presoaking with various concentrations of WV. The volum ratio[primary WV:ddH2O2 (V:V)] is 0 (control), 1:300, 1:600, 1:900, 1:1200, and 1:1500 from left to right. D, C, the analysis of roots system of the control and WV-treated(1:900) under drought stress.

## Slide 3
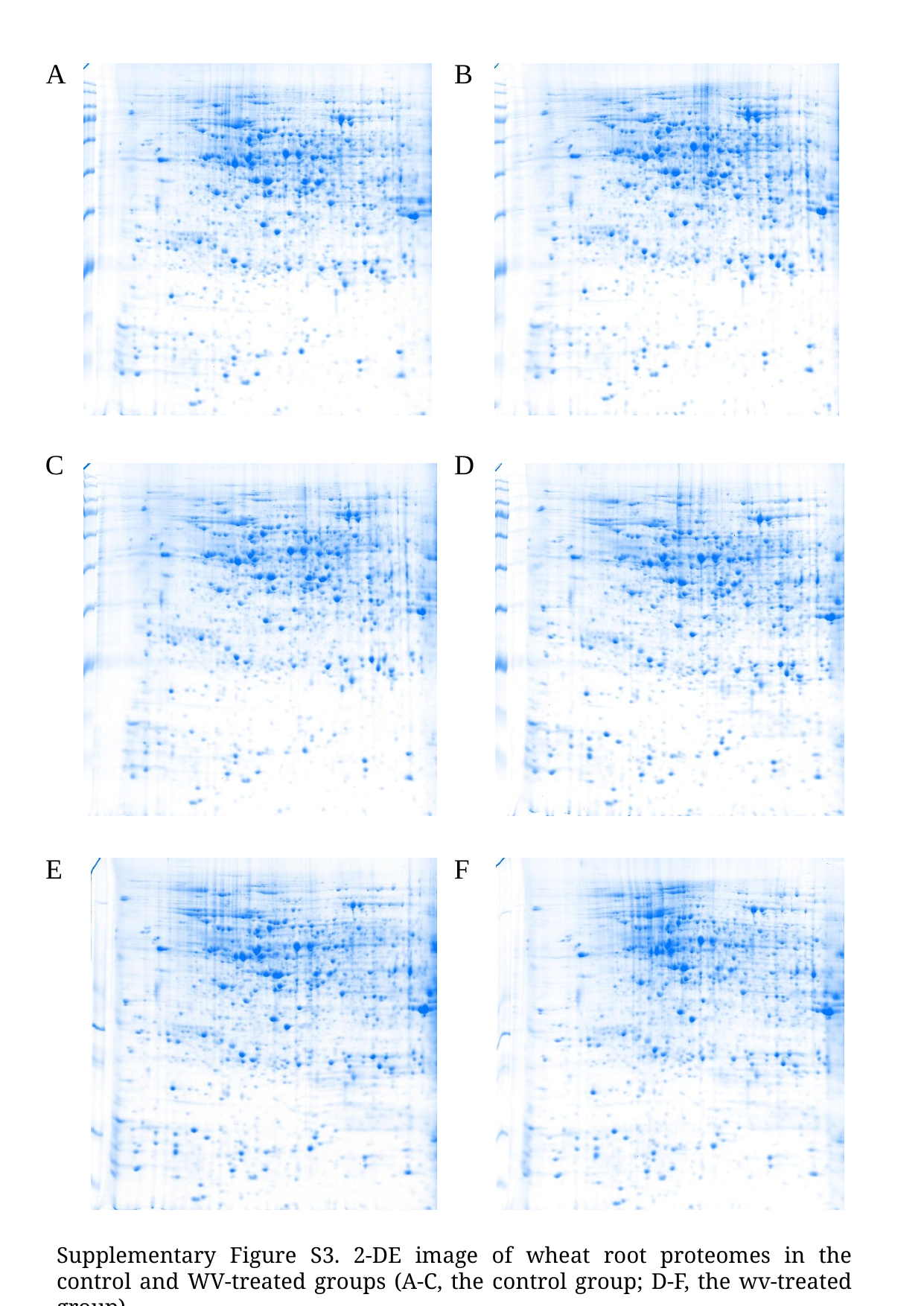

A
B
C
D
E
F
Supplementary Figure S3. 2-DE image of wheat root proteomes in the control and WV-treated groups (A-C, the control group; D-F, the wv-treated group)

## Slide 4
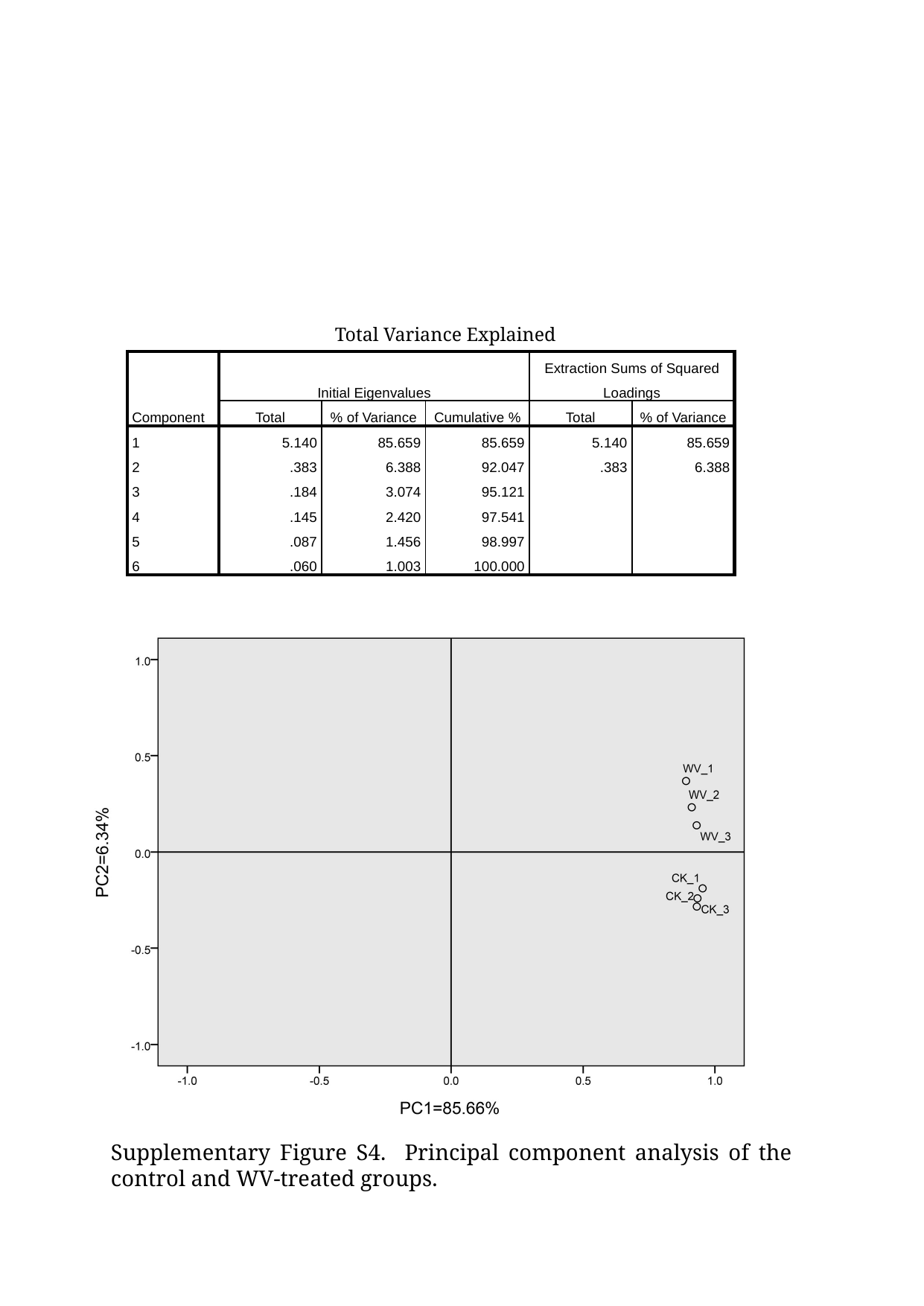

Total Variance Explained
| Component | Initial Eigenvalues | | | Extraction Sums of Squared Loadings | |
| --- | --- | --- | --- | --- | --- |
| | Total | % of Variance | Cumulative % | Total | % of Variance |
| 1 | 5.140 | 85.659 | 85.659 | 5.140 | 85.659 |
| 2 | .383 | 6.388 | 92.047 | .383 | 6.388 |
| 3 | .184 | 3.074 | 95.121 | | |
| 4 | .145 | 2.420 | 97.541 | | |
| 5 | .087 | 1.456 | 98.997 | | |
| 6 | .060 | 1.003 | 100.000 | | |
Supplementary Figure S4. Principal component analysis of the control and WV-treated groups.

## Slide 5
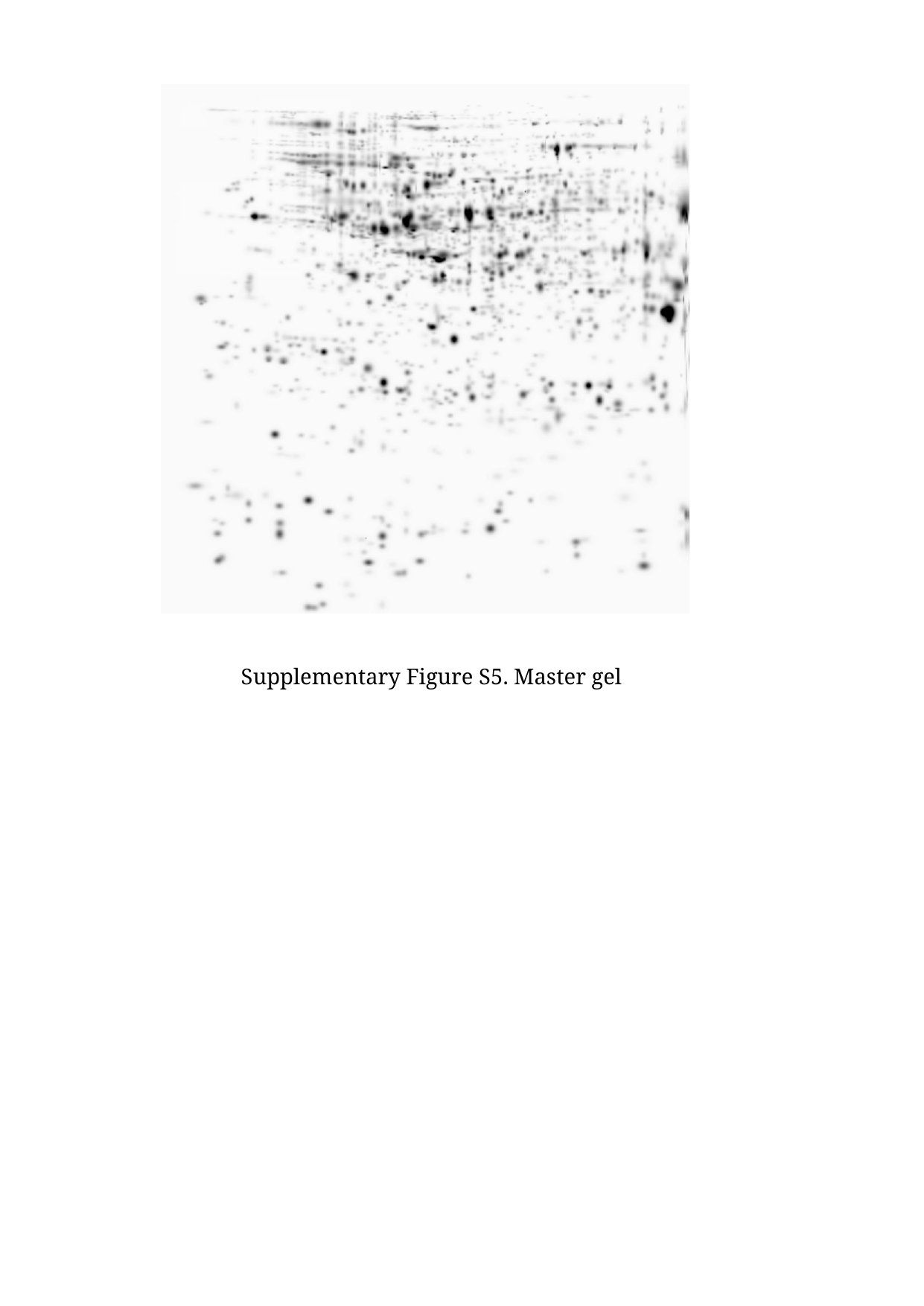

Supplementary Figure S5. Master gel

## Slide 6
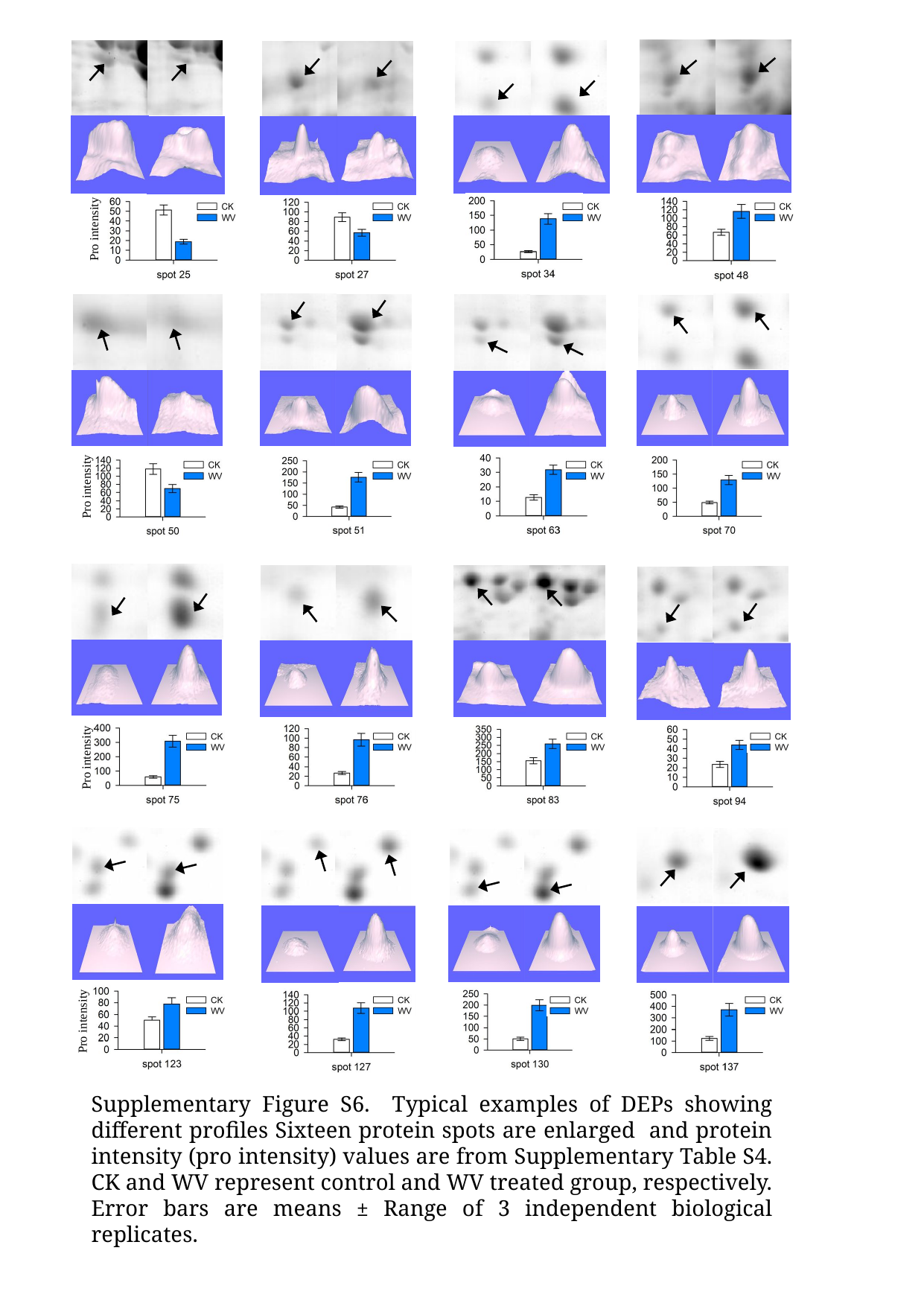

Pro intensity
Pro intensity
Pro intensity
Pro intensity
Supplementary Figure S6. Typical examples of DEPs showing different profiles Sixteen protein spots are enlarged and protein intensity (pro intensity) values are from Supplementary Table S4. CK and WV represent control and WV treated group, respectively. Error bars are means ± Range of 3 independent biological replicates.

## Slide 7
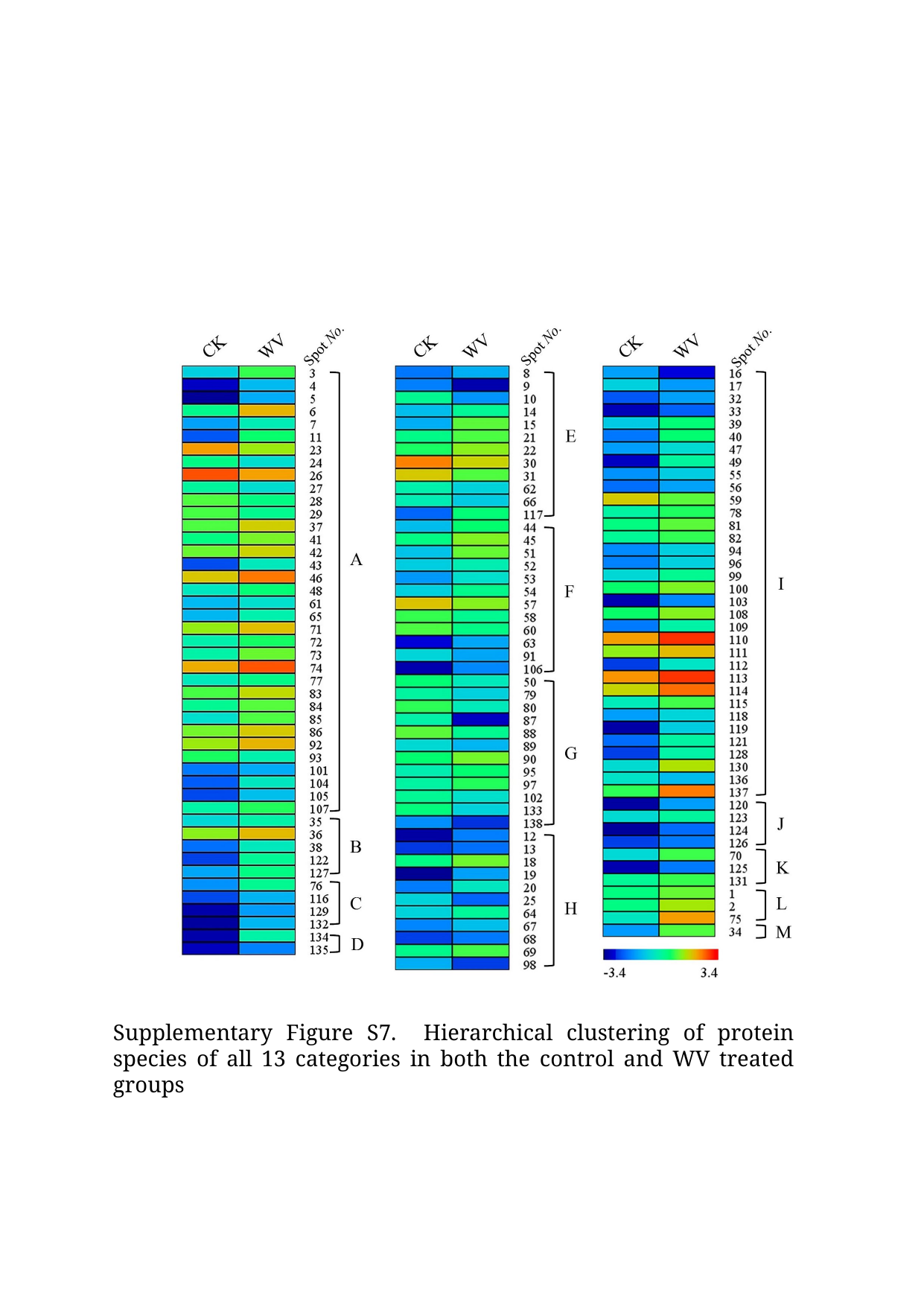

Supplementary Figure S7. Hierarchical clustering of protein species of all 13 categories in both the control and WV treated groups

## Slide 8
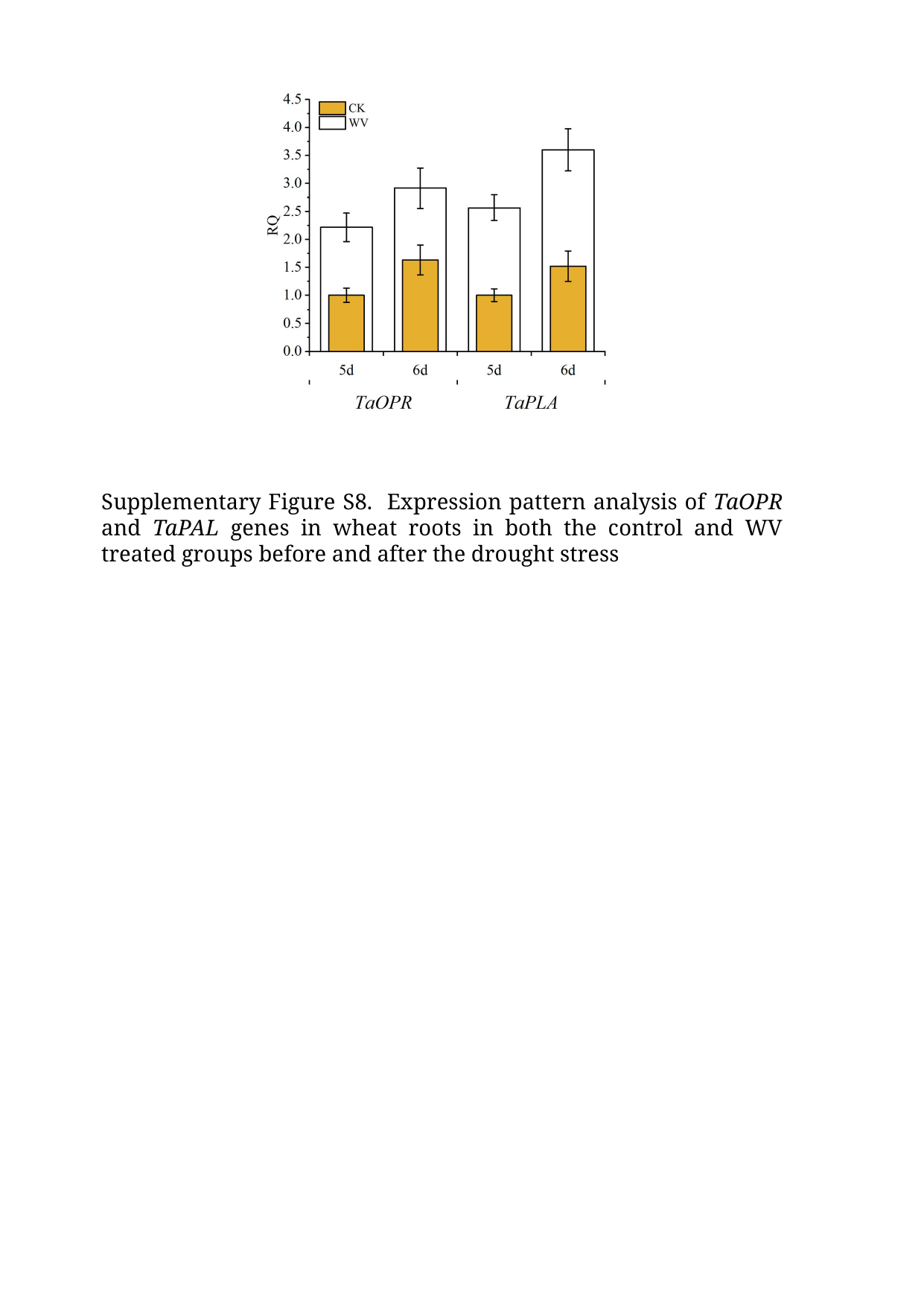

Supplementary Figure S8. Expression pattern analysis of TaOPR and TaPAL genes in wheat roots in both the control and WV treated groups before and after the drought stress
