# Supplementary figures and images for: Root Proteomics Reveals the Effects of Wood Vinegar on Wheat Growth and Subsequent Tolerance to Drought Stress
Source: Int J Mol Sci. 2019 Feb 21;20(4):943. doi: 10.3390/ijms20040943 (PMC6413028; doi:10.3390/ijms20040943)

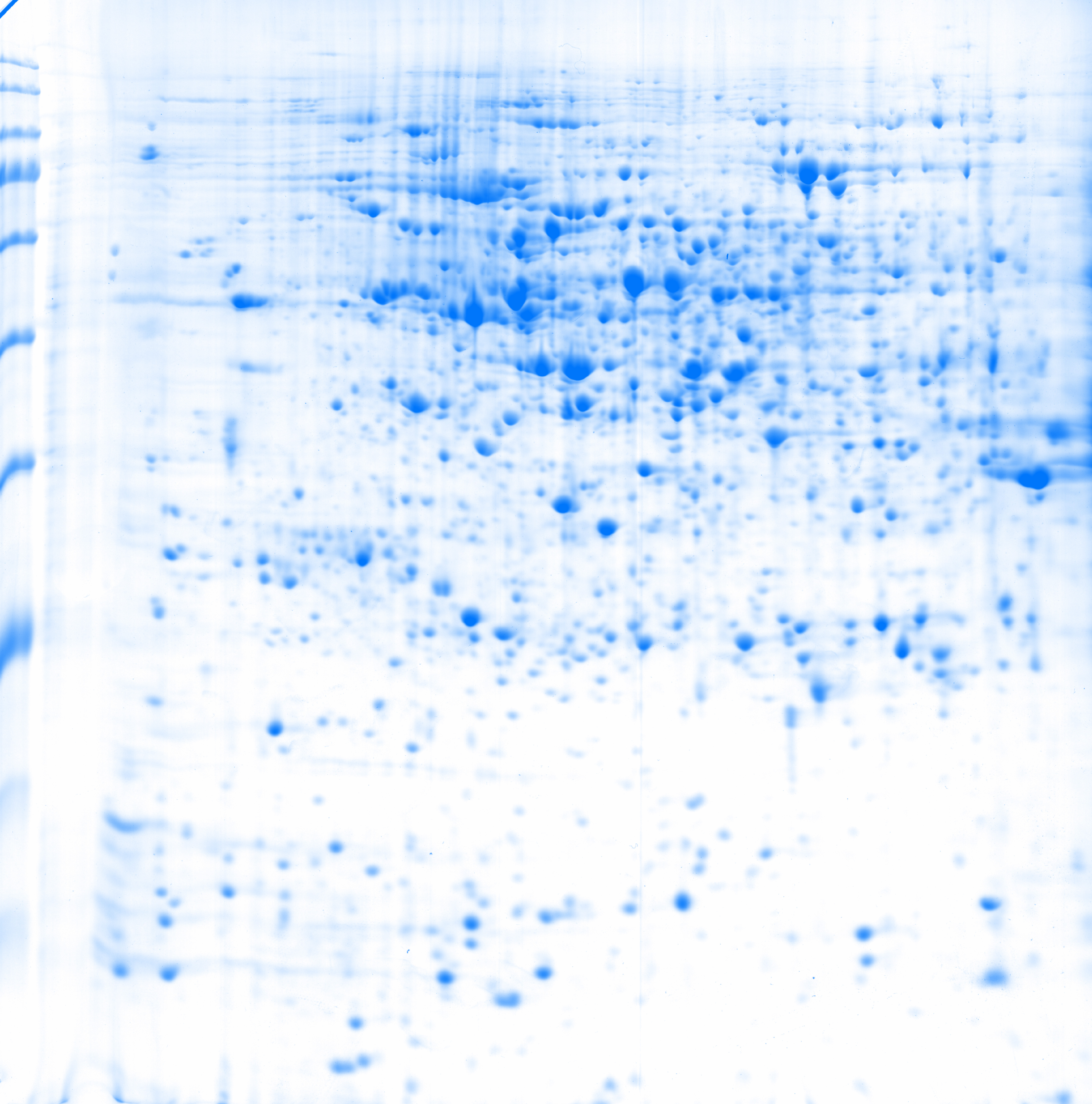

Supplement: Supplementary file 1 [file ijms-20-00943-s001.zip › proofed version_ijms-449598-supplementary/Supplementary Figure S3/CK-1.jpg]

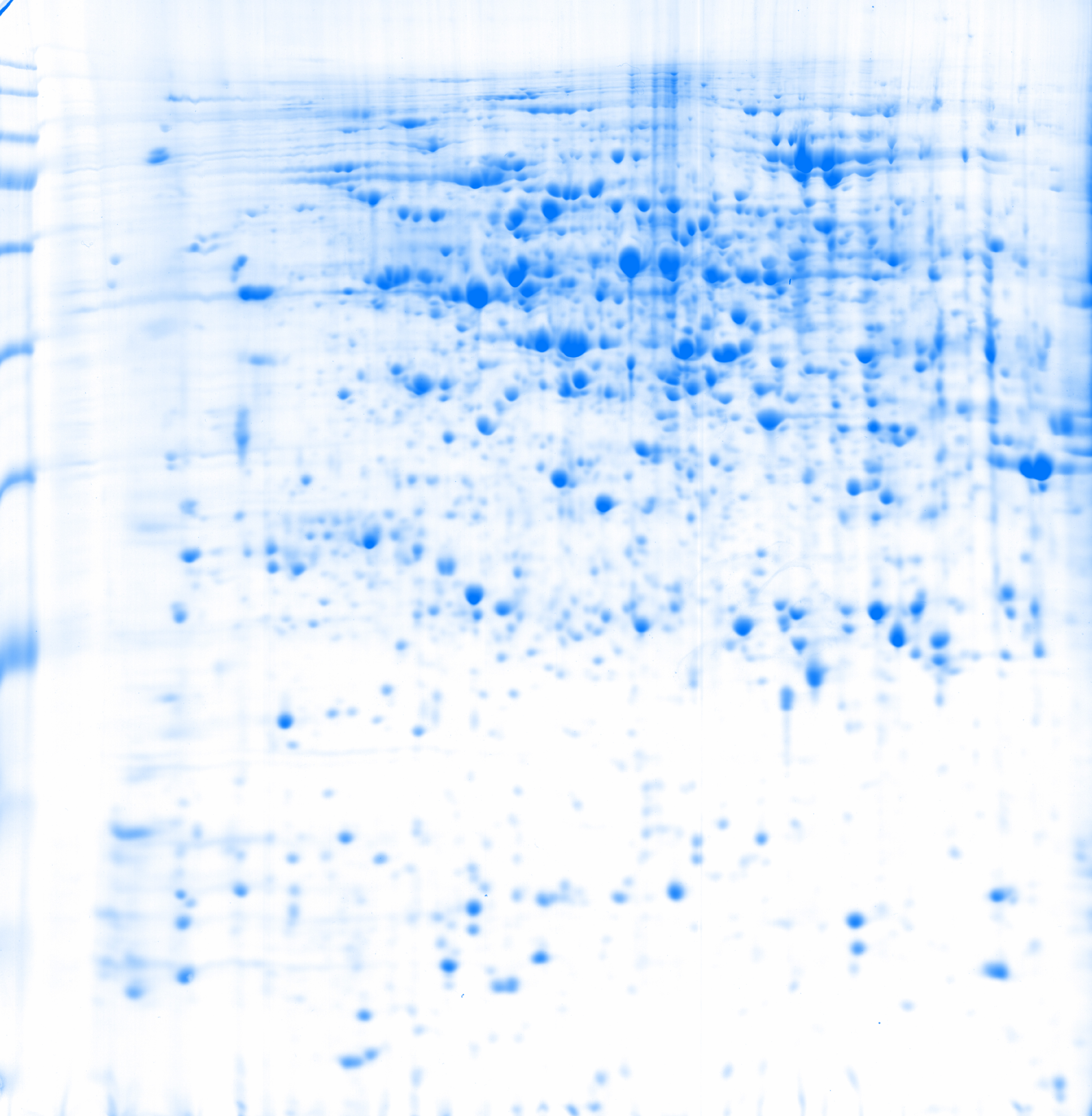

Supplement: Supplementary file 1 [file ijms-20-00943-s001.zip › proofed version_ijms-449598-supplementary/Supplementary Figure S3/CK-2.jpg]

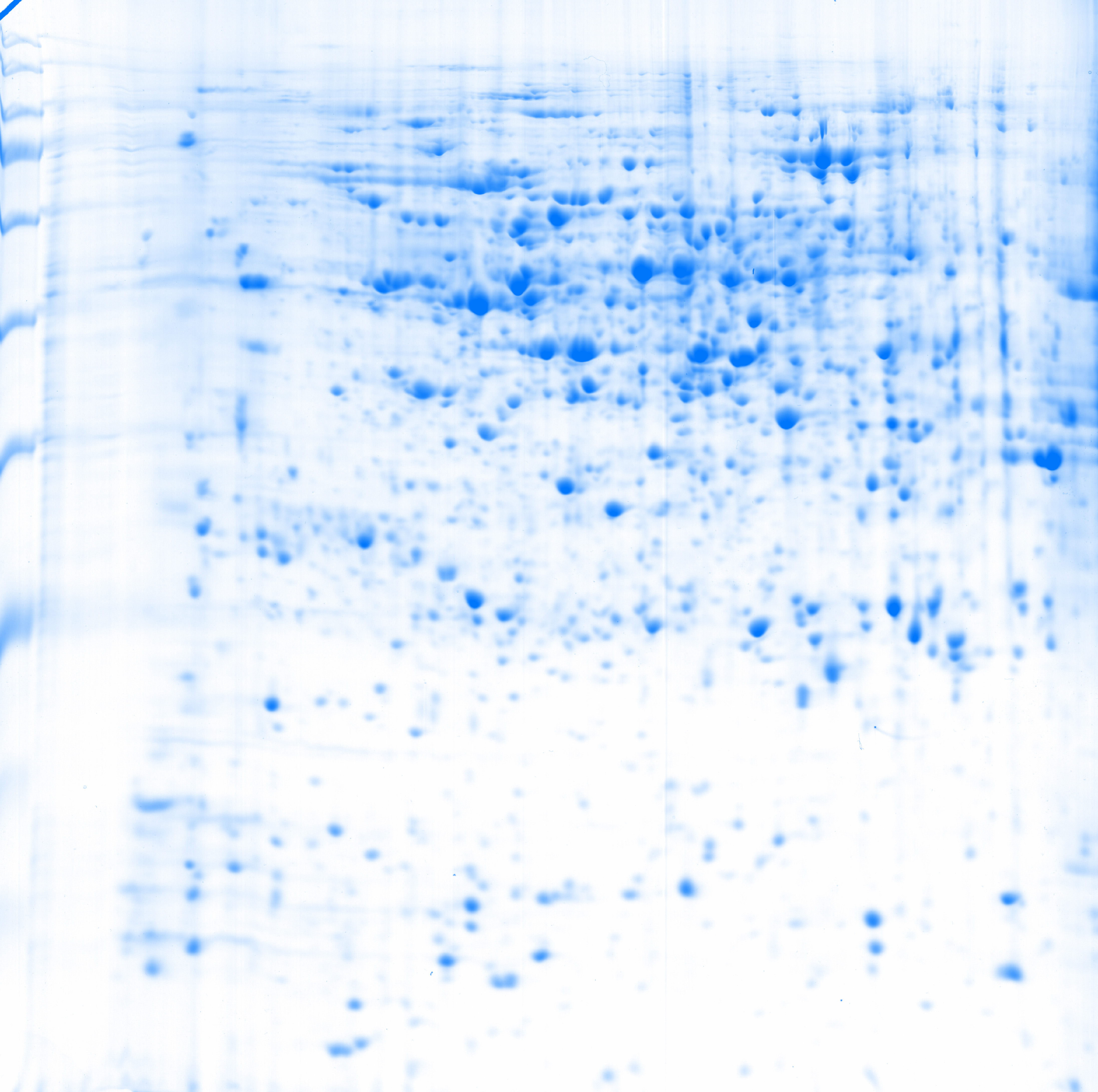

Supplement: Supplementary file 1 [file ijms-20-00943-s001.zip › proofed version_ijms-449598-supplementary/Supplementary Figure S3/CK-3.jpg]

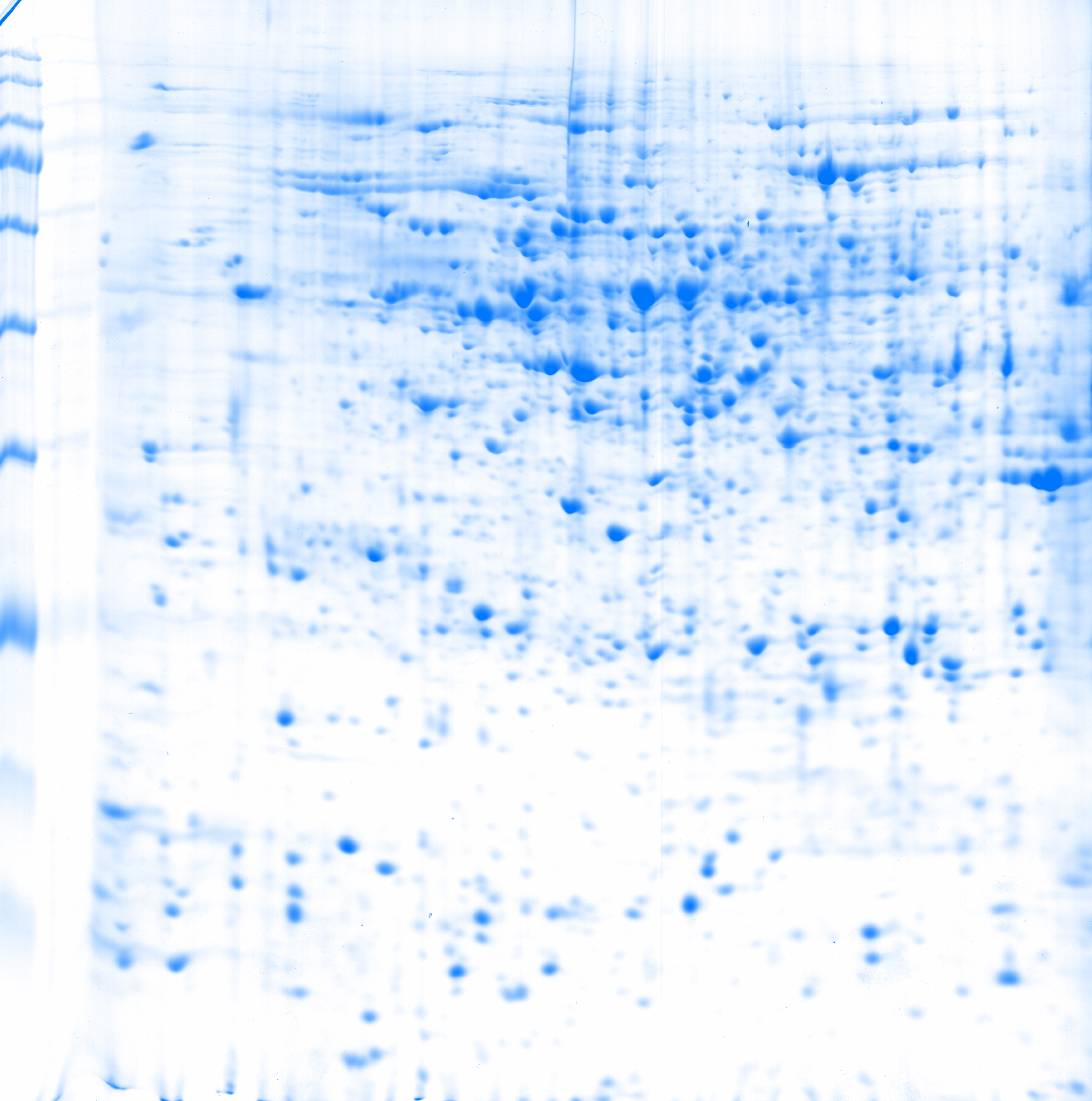

Supplement: Supplementary file 1 [file ijms-20-00943-s001.zip › proofed version_ijms-449598-supplementary/Supplementary Figure S3/WV-1.jpg]

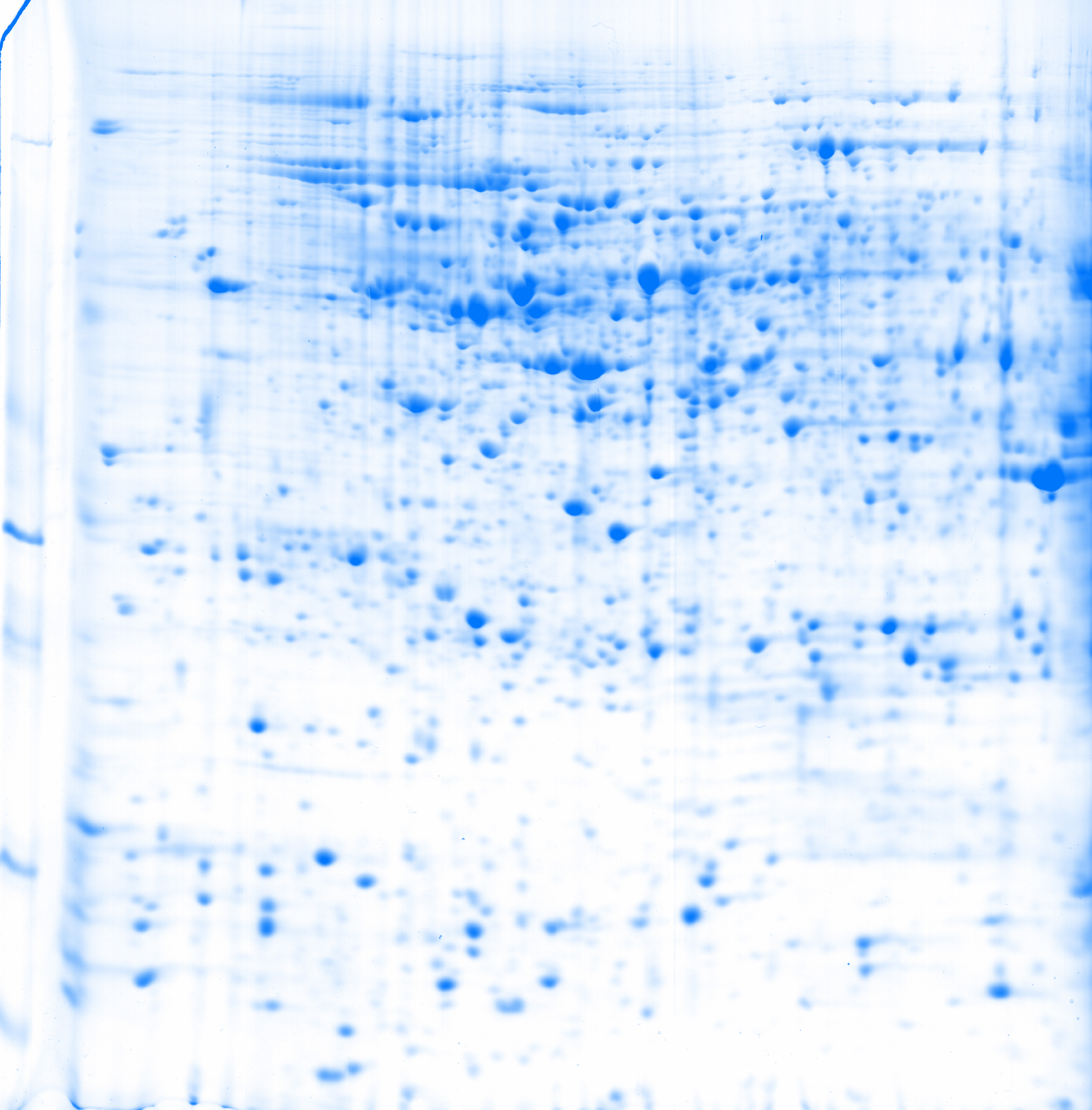

Supplement: Supplementary file 1 [file ijms-20-00943-s001.zip › proofed version_ijms-449598-supplementary/Supplementary Figure S3/WV-2.jpg]

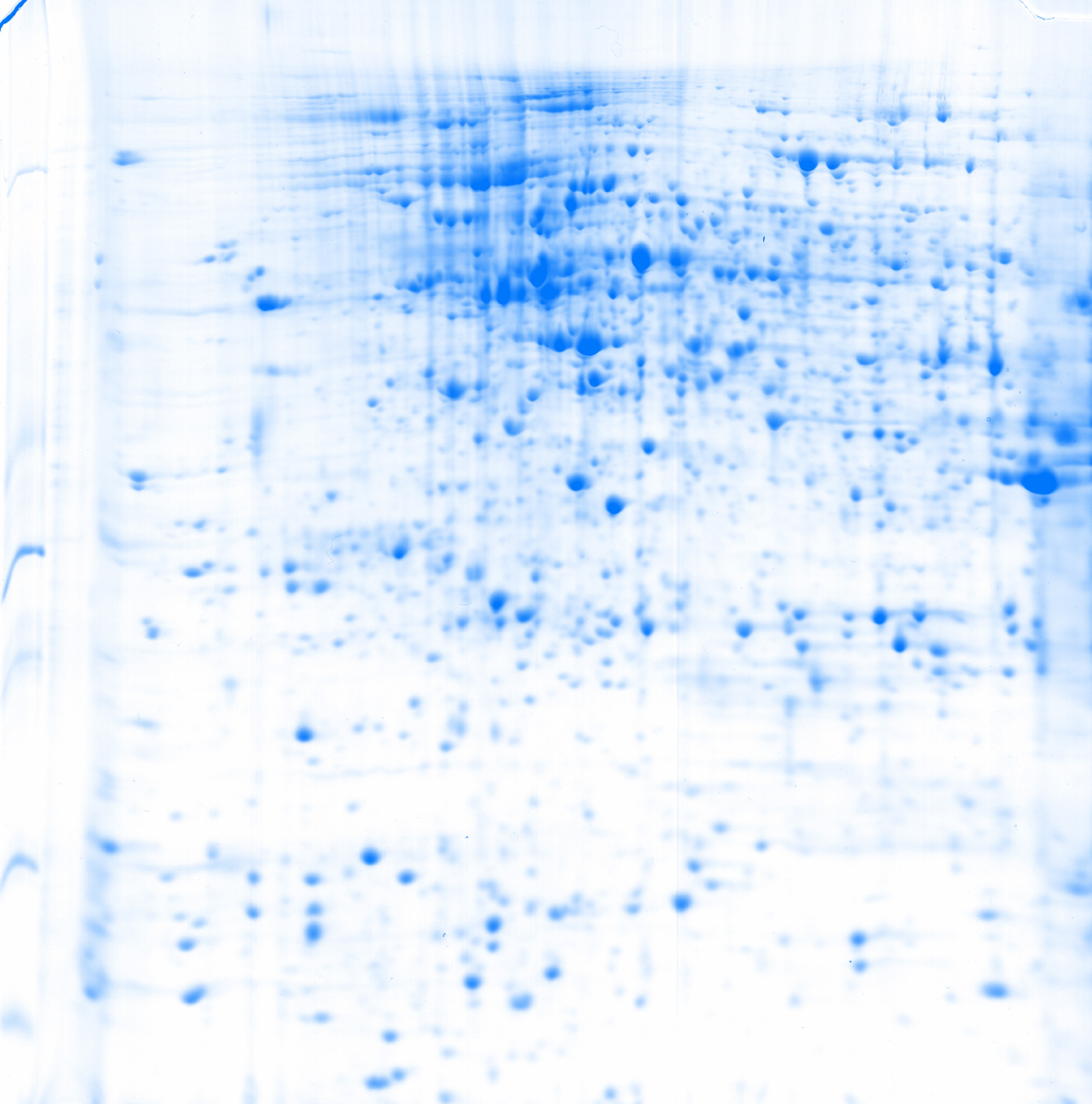

Supplement: Supplementary file 1 [file ijms-20-00943-s001.zip › proofed version_ijms-449598-supplementary/Supplementary Figure S3/WV-3.jpg]

CK WV (Master)

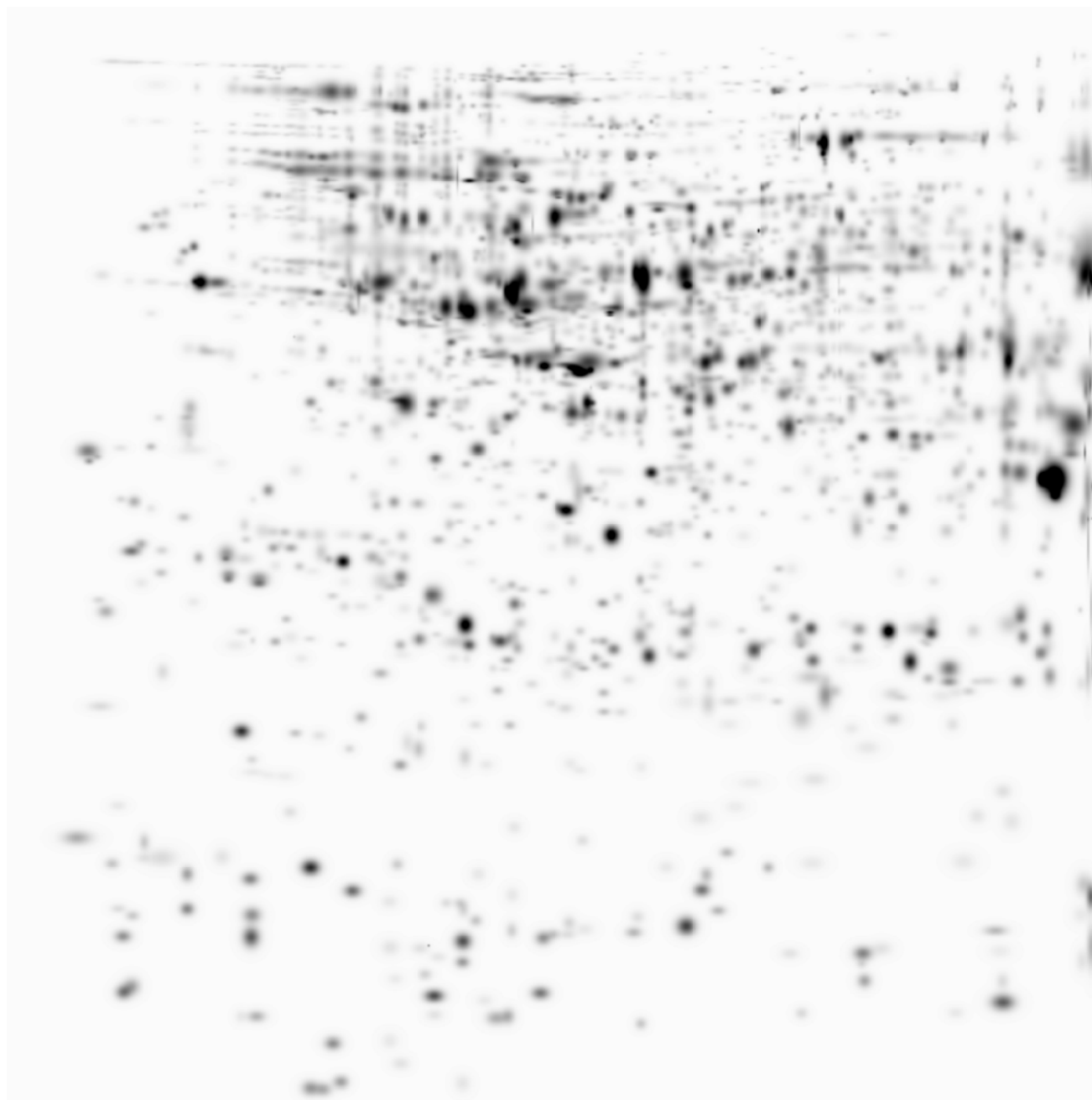

Supplement: Supplementary file 1 [file ijms-20-00943-s001.zip › proofed version_ijms-449598-supplementary/Supplementary Figure S5.pdf]

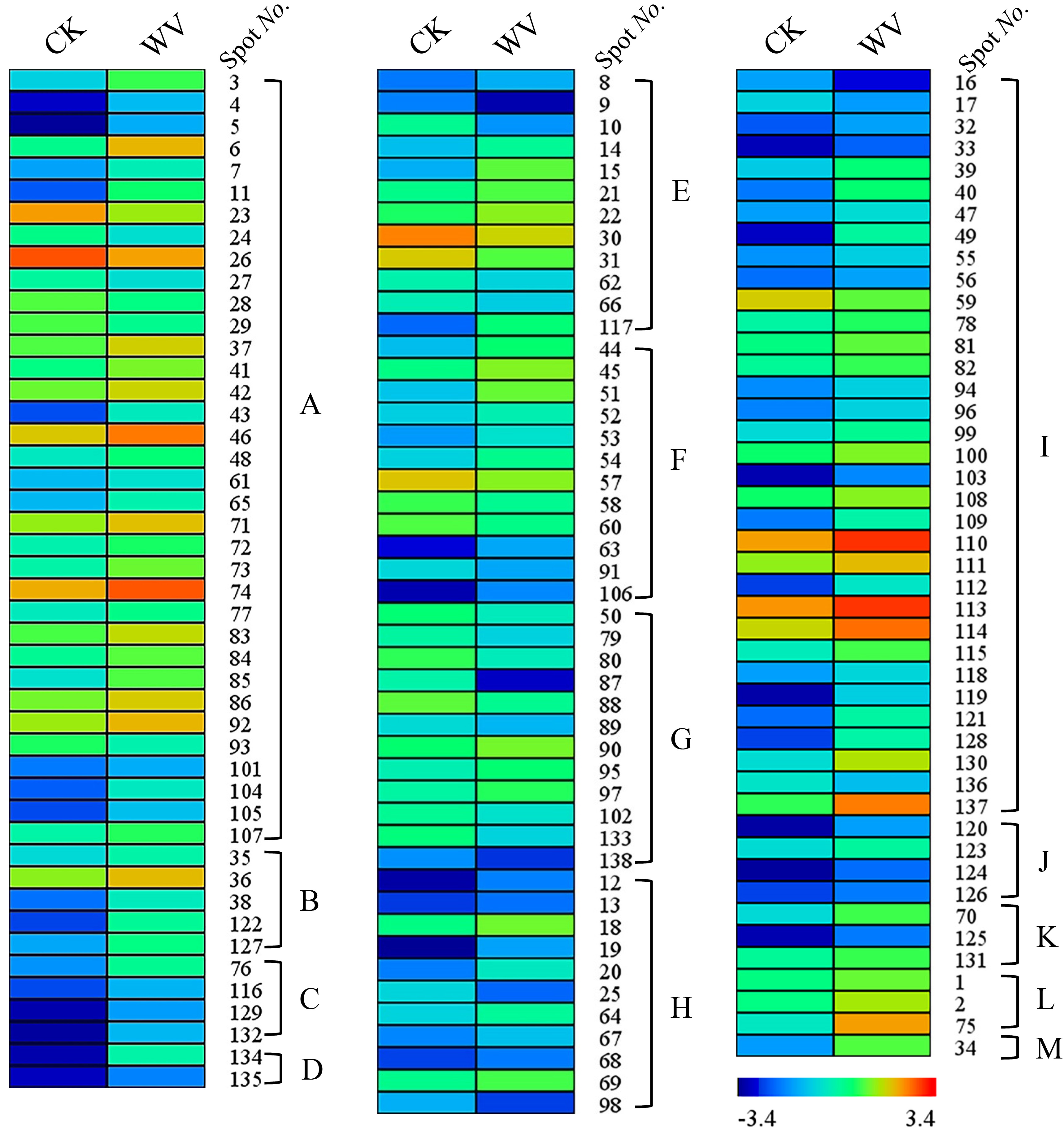

Supplement: Supplementary file 1 [file ijms-20-00943-s001.zip › proofed version_ijms-449598-supplementary/Supplementary Figure S7.jpg]

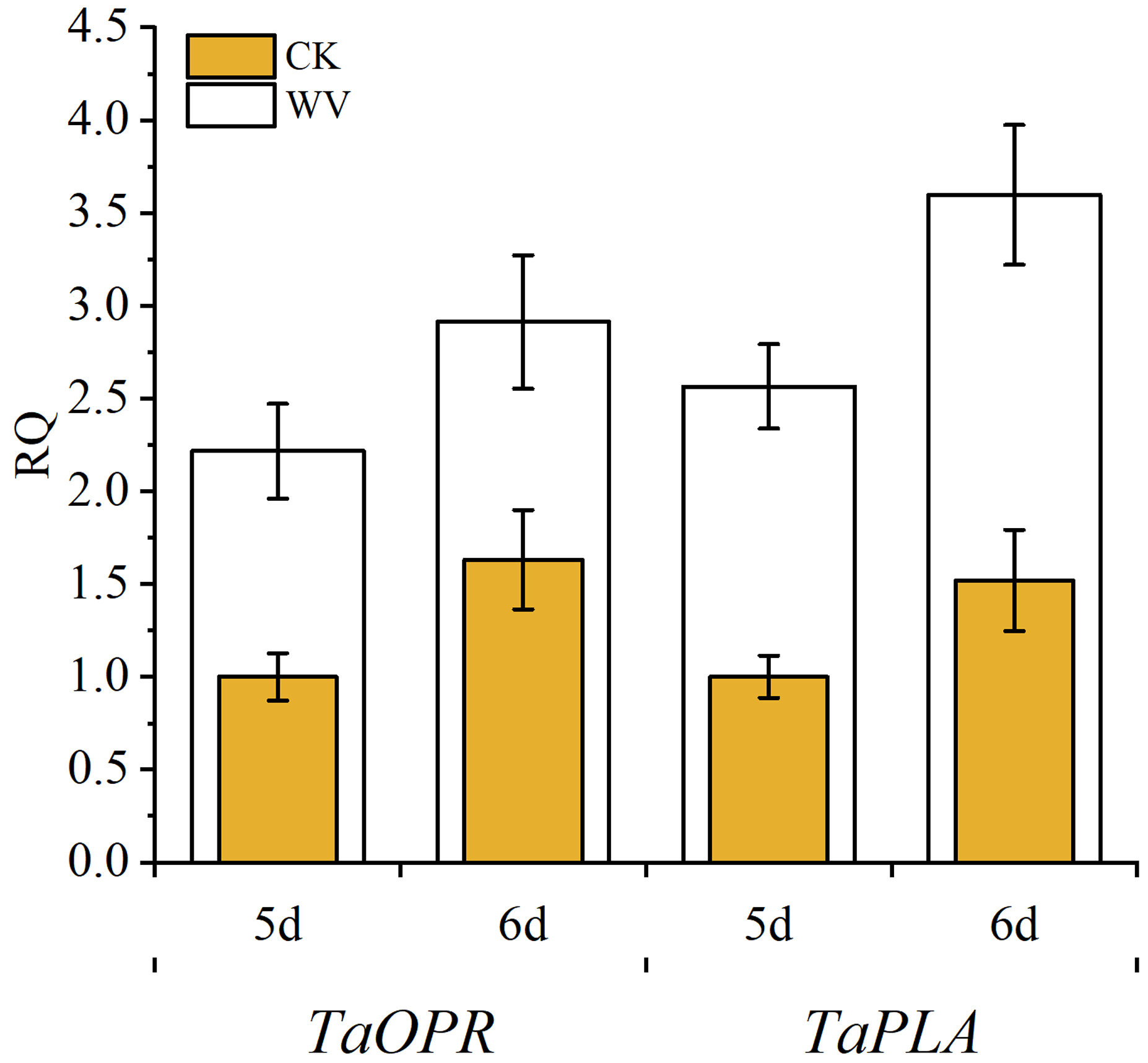

Supplement: Supplementary file 1 [file ijms-20-00943-s001.zip › proofed version_ijms-449598-supplementary/Supplementary Figure S8.tif]
